# Single Nucleus Genome Sequencing Reveals High Similarity among Nuclei of an Endomycorrhizal Fungus

**DOI:** 10.1371/journal.pgen.1004078

**Published:** 2014-01-09

**Authors:** Kui Lin, Erik Limpens, Zhonghua Zhang, Sergey Ivanov, Diane G. O. Saunders, Desheng Mu, Erli Pang, Huifen Cao, Hwangho Cha, Tao Lin, Qian Zhou, Yi Shang, Ying Li, Trupti Sharma, Robin van Velzen, Norbert de Ruijter, Duur K. Aanen, Joe Win, Sophien Kamoun, Ton Bisseling, René Geurts, Sanwen Huang

**Affiliations:** 1 Laboratory of Computational Molecular Biology, College of Life Sciences, Beijing Normal University, Beijing, China; 2 Laboratory of Molecular Biology, Department of Plant Science, Wageningen University, Wageningen, The Netherlands; 3 Institute of Vegetables and Flowers, Chinese Academy of Agricultural Sciences, Key Laboratory of Biology and Genetic Improvement of Horticultural Crops of Ministry of Agriculture, Sino-Dutch Joint Lab of Horticultural Genomics, Beijing, China; 4 The Sainsbury Laboratory, Norwich Research Park, Norwich, United Kingdom; 5 Novome Biotech Inc., Zhongguancun Life Science Park, Beijing, China; 6 Laboratory of Cell Biology, Department of Plant Science, Wageningen University, Wageningen, The Netherlands; 7 Laboratory of Genetics, Department of Plant Science, Wageningen University, Wageningen, The Netherlands; 8 College of Science, King Saud University, Riyadh, Saudi Arabia; 9 Agricultural Genome Institute at Shenzhen, Chinese Academy of Agricultural Sciences, Shenzhen, China; University College Dublin, Ireland

## Abstract

Nuclei of arbuscular endomycorrhizal fungi have been described as highly diverse due to their asexual nature and absence of a single cell stage with only one nucleus. This has raised fundamental questions concerning speciation, selection and transmission of the genetic make-up to next generations. Although this concept has become textbook knowledge, it is only based on studying a few loci, including 45S rDNA. To provide a more comprehensive insight into the genetic makeup of arbuscular endomycorrhizal fungi, we applied *de novo* genome sequencing of individual nuclei of *Rhizophagus irregularis*. This revealed a surprisingly low level of polymorphism between nuclei. In contrast, within a nucleus, the 45S rDNA repeat unit turned out to be highly diverged. This finding demystifies a long-lasting hypothesis on the complex genetic makeup of arbuscular endomycorrhizal fungi. Subsequent genome assembly resulted in the first draft reference genome sequence of an arbuscular endomycorrhizal fungus. Its length is 141 Mbps, representing over 27,000 protein-coding gene models. We used the genomic sequence to reinvestigate the phylogenetic relationships of *Rhizophagus irregularis* with other fungal phyla. This unambiguously demonstrated that Glomeromycota are more closely related to Mucoromycotina than to its postulated sister Dikarya.

## Author Summary

Endomycorrhizal fungi are known for their symbiosis with the vast majority of land plants. The fungus penetrates the root and facilitates uptake of nutrients for the plant. For a long time it was hypothesized that endomycorrhizal fungi have a complex genetic makeup, as they are asexual organisms. Their hyphae do not consist of individual cells, but rather form a continuous compartment in which numerous nuclei migrate. Several studies indicated that these nuclei are genetically highly diverse, suggesting that endomycorrhizal fungi evolved a unique genome structure. By sequencing individual nuclei of a single individual of the reference fungus Rhizophagus, we demystify this hypothesis and show that the nuclei are highly similar. Furthermore, we created the first genome sequence of these ancient fungi that will serve as a valuable resource to further understand and exploit this agriculturally and ecologically vital symbiosis.

## Introduction

The interaction of arbuscular endomycorrhizal (AM) fungi and land plants is a very successful symbiosis as it is ancient (∼450 million years), and maintained by the vast majority of plant species [1]. AM fungi are obligate biotrophs that infect roots and form highly branched structures (arbuscules) inside root cortical cells [1]. These arbuscules are connected to an extensive network of extraradical mycelium that facilitates uptake of nutrients from the soil, e.g. immobile phosphates.

AM hyphal networks form a continuous coenocytic compartment with numerous nuclei. AM fungi are considered to be ancient asexual organisms [2]–[4] and propagation occurs via spores that become filled with multiple nuclei that subsequently divide [5]. AM fungal individuals can be heterokaryotic, i.e. consist of genetically divergent nuclei, because single nucleus cellular stages never occur during the lifecycle, and because hyphae of different fungal individuals can fuse and exchange nuclei by anastomosis [6], [7]. Our knowledge of the genome structure of AM fungi is rudimentary. For instance, the degree to which a minimal gene set is present in a single nucleus, or is distributed over genetically distinct nuclei is unknown [2], [8]–[11]. Although there is evidence for genetic variability within single spores, the genomic organization of this variation remains elusive. Two competing hypotheses have been advocated. The genetic variation may be present in a single, possibly polyploid, nucleus [9], or it could be distributed over multiple nuclei in a single individual [8], [10]. However, in reality these hypotheses may represent extremes along a continuum of genetic variation among and within nuclei [2].

Extensive efforts to sequence the genome of the reference AM fungal species *Rhizophagus irregularis* DAOM197198 (previously known as *Glomus intraradices* [12], [13] have not been successful, possibly because of its heterokaryotic nature [14]. To address this issue and determine the extent to which nuclei are indeed markedly different, we conducted *de novo* genome sequencing of individual nuclei of an *R. irregularis* line isolated from the reference strain DAOM197198 (designated DAOM197198w). The resulting *R. irregularis* genome sequence revealed a surprisingly low level of polymorphism between nuclei.

## Results and Discussion

### Genome sequencing of individual nuclei reveals that *R. irregularis* is homokaryotic

**Figure 1:**
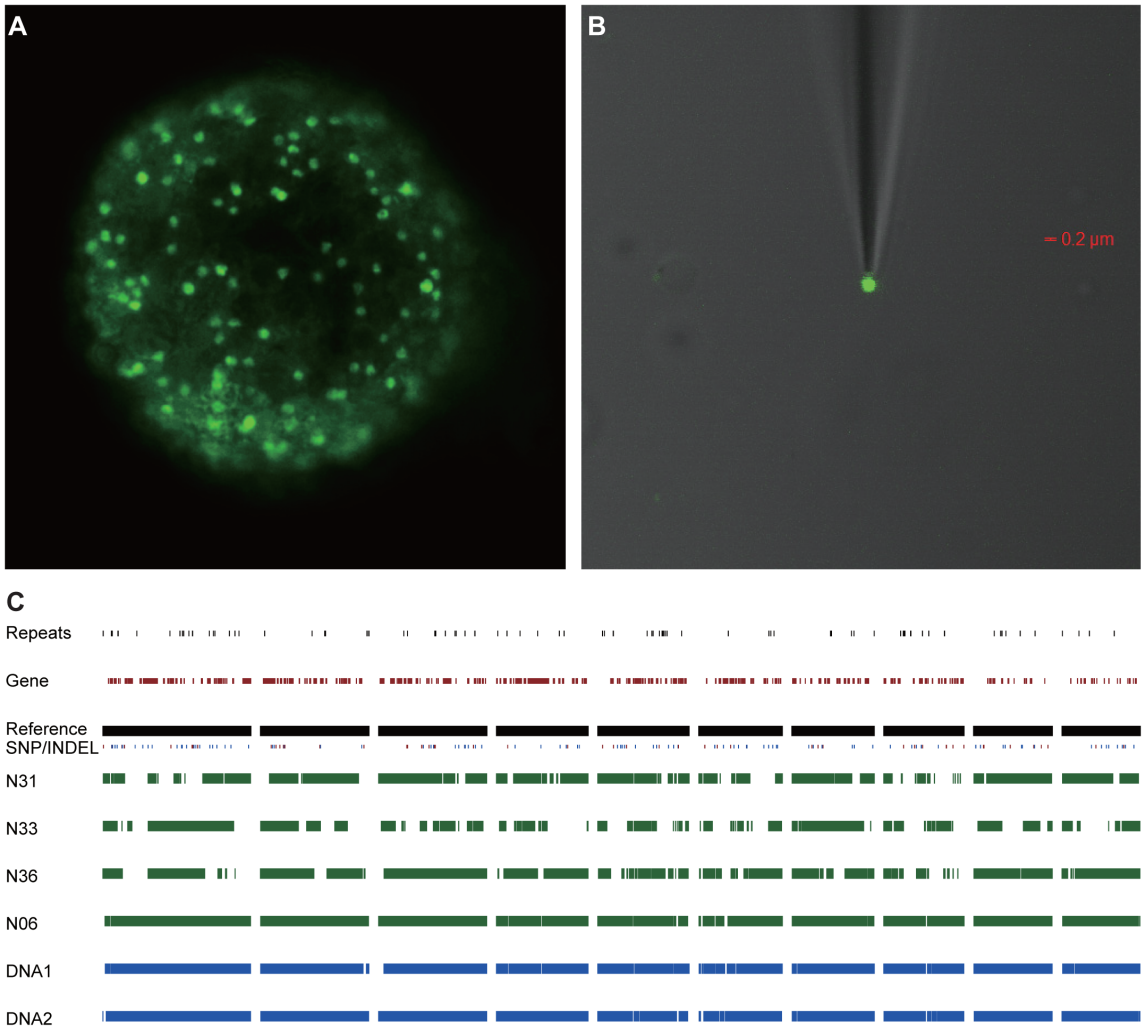
Genome sequence of single *R. irregularis* DAOM197198w nuclei. (**A**) Sytox Green stained spore containing numerous nuclei. (**B**) Single Sytox-stained nucleus trapped with a micropipette. (**C**) Level of homology between four individual nuclei (N6, N31, N33 and N36) and 2 mycelium DNA samples (DNA1 and DNA2). Presented are the 10 largest contigs of the reference genome (representing ∼1,278 kb). The occurrence of SNPs (marked in blue) and INDELs (marked in red), and gene distributions, in the different assemblies are indicated.

Spores of a mycorrhized root culture of chicory (*Cichorium intybus*) were stained by 10 µM Sytox Green (Fig. 1A). Single nuclei were collected from a supernatant of crushed spores using a micromanipulator (Fig. 1B). Individual nuclei were immediately processed for whole genome amplification. To verify the quality of the amplified nuclear DNA ten randomly selected loci were PCR amplified, and also the extent of bacterial contamination was monitored. Four amplified single nucleus genomes were processed for sequencing, resulting in assembled genomes of 115, 90, 71 and 95 Mbps, respectively (Tables S1 and S2). The different sizes of the assemblies are likely reflecting variation in the whole genome amplification efficiencies among the four samples. First comparative analyses detected surprisingly few SNPs and indels across the four nuclei. This suggested that nuclei are markedly more similar than was expected. Therefore we decided to sequence also two DNA samples extracted from mycelium. The generated sequences of these DNA samples (designated DNA1 and DNA2) were assembled individually, resulting in genome assemblies of 116 and 117 Mbps, respectively (Table 1). Additionally, the six genome sequences were assembled together resulting in a reference genome for *R. irregularis* of 141 Mbps. A self-alignment of this reference genome revealed little redundancy ruling out the occurrence of (significant) artificial duplifications within the assembly (Fig. S1). By comparative genomic analysis, only 28,872 SNPs and 12,315 indels were detected across the six assemblies when compared to the reference genome (Fig. 1C, Table S2). Furthermore, a reference-independent comparison of the four single nuclei and the two mycelial samples also revealed a comparable low level of polymorphisms (Table S3). This indicates that more than 99.97% of the (aligned) genome sequence is identical between different nuclei. Furthermore, as the size of the assembled genome is in line with previous estimates of the DNA content of nuclei [15], we conclude that *R. irregularis* nuclei are haploid.

**Table 1:** Characteristics of the seven genome assemblies from *R. irregularis* DAOM197198w.

		Reference	DNA1	DNA2	N6	N31	N33	N36
Contigs	N20 (bp)	35,093	41,795	43,394	46,162	23,481	18,221	25,221
	N50 (bp)	16,014	18,598	19,912	19,648	10,530	8,434	11,121
	N80 (bp)	4,077	6,103	6,420	6,341	3,678	3,065	3,795
	Av. length (bp)	2,366	1,684	1,620	1,544	2,309	2,330	2,269
	Max. length (bp)	189,408	208,614	214,169	171,410	79,274	61,908	107,227
	Total number	31,773	14,093	13,797	13,787	16,493	15,073	16,893
	Total (Mb)	140.5	115.8	117.1	115	90.4	71.6	95.5
Scaffolds	N20 (bp)	35,747	43,804	45,734	48,447	24,016	18,834	26,344
	N50 (bp)	16,360	19,381	20,888	20,759	10,982	8,624	11,688
	N80 (bp)	4,293	6,331	6,752	6,713	3,813	3,144	3,935
	Av. length (bp)	2,322	1,599	1,532	1,470	2,223	2,265	2,175
	Max. length (bp)	198,933	208,614	214,169	171,626	79,274	69,834	107,227
	Total number	30,638	13,333	12,871	12,603	15,672	14,550	15,949
	Total (Mb)	140.9	115.9	117.1	115	90.4	71.6	95.5

N50: the length for which the contigs (scaffolds) of that length or longer contains at least half of the total lengths of the contigs (scaffolds).

**Figure 2:**
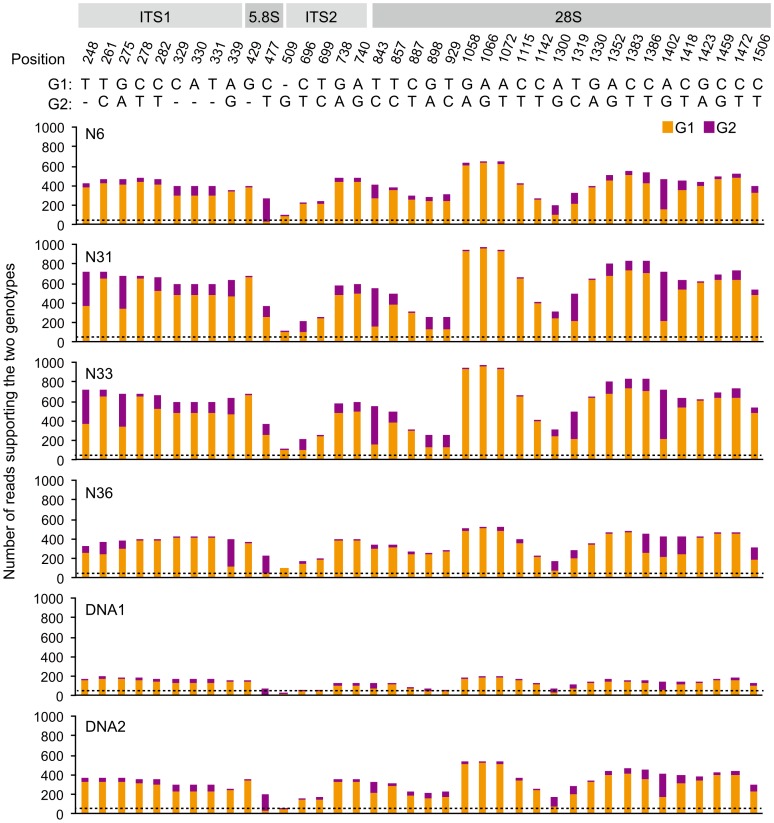
Overview of polymorphisms in the *R. irregularis* 45S rDNA repeat unit in four individual nuclei. The top part indicates the various regions within the *R. irregularis* DAOM197198 45S rDNA reference sequence (Genbank JF439109). Position means the position of each polymorphic site on the reference. G1: genotype identical to reference; G2: polymorphic nucleotide. The six histograms show the numbers of sequenced reads supporting the two genotypes for N6, N31, N33, N36 and mycelium DNA samples DNA1 and DNA2. The dashed lines indicate the average sequencing depth for each sample.

Several loci have previously been used to determine genetic polymorphisms within AM individuals. These include *Binding Protein (BIP)*, SSR marker *Bg112*, the internal transcribed spacers (ITS1 and ITS2) of the 45S rDNA locus in *R. irregularis* and *POL1-Like Sequence* (*PLS*) in *Glomus etunicatum* [8], [9], [16]. We compared these loci in the different genome assemblies. Only a single *PLS* homolog was identified in *R. irregularis* (*RiPLS*, RirG174000), whereas *G. etunicatum* has multiple copies that belong to two main types, of which the highly polymorphic *PLS1* likely represents a pseudogene [9], [17]. No polymorphisms were found for *RiPLS* in the different assemblies (Fig. S2). For *BIP* three loci were identified and designated *RiBIP1* (RirG196040), *RiBIP2* (RirG160690) and *RiBIP3* (RirG043980). Sequence and structure of these genes is highly conserved and homologous to a *Rhizopus delemar* 70 kD Heat shock protein (GenBank: EIE83965). *RiBIP1*, *RiBIP2* and *RiBIP3* are present also in nucleus 6 without allelic variation when compared to the DNA1 and DNA2 genome assemblies. This holds true also for the other three sequenced nuclei, though not all three *BIP* loci were covered in the genome assemblies, which can be attributed to incomplete amplification (Fig. S3). Next, we studied *Bg112* for which three loci were identified. Again, no allelic variation was detected among the four nuclei (Fig. S4). The polymorphism of the ITS region of the multi-copy 45S rDNA locus was studied within each of the 4 nuclei. By mapping sequence reads to a reference *R. irregularis* ITS sequence (Genbank JF439109), many variants reported previously for strain DAOM197198 were identified within individual nuclei (Fig. 2) [8], [12]. This demonstrates that, in addition to reported intraspecific ITS variability within single *R. irregularis* spores [12], [18], the ITS region in the multi-repeat 45S rDNA locus is extremely variable even within individual nuclei, and that different nuclei can show quantitative variation in polymorphic ITS variants. In general, multi-repeat loci such as rDNA sequences are thought to be homogenized through concerted evolution [19], which presumably is most effective during meiosis [20], [21]. Therefore, the high level of heterogeneity among the copies within a single repeat seems to be consistent with ancient asexuality. However, also in several sexual fungal species varying levels of intra-individual polymorphism have been found [22], and *R. irregularis* may be an extreme case, although exact percentages cannot be deduced from the Illumina read data. Given the high level of ITS variability within single nuclei, we conclude that the 45rDNA ITS sequence is less suited for comparative studies of Glomeromycota. Based on the whole genome comparison of individual nuclei we conclude that the organization of the *R. irregularis* genome of the used reference culture DAOM197198w is basically homokaryotic. The high divergence observed among copies of the 45S rDNA repeat occurs within a single nucleus, indicating that this region is unsuited to claim that nuclei within a strain are highly divergent [8]. However, the presence of a low level of polymorphisms suggests that genetically, slightly divergent nuclei can arise and coexist in a single mycelium.

### Genetic make-up of *R. irregularis*

**Figure 3:**
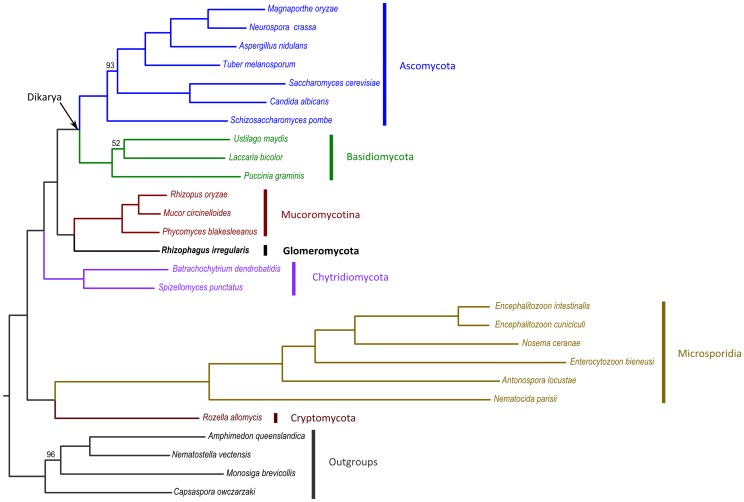
ML tree derived from the concatenation of 35 widespread, single-copy genes. The amino acid alignment was trimmed as explained in the Materials and methods section to remove non-informative positions, resulting in 26,604 positions. The tree was estimated using the rtREV evolutionary model implemented in RAxML. Bootstrap analysis was performed based on 100 replicates, and the three nodes with support below 100 are indicated. Scale bar indicates average number of amino acid substitutions per site.

The reference genome assembly of DAOM197198w covers about 97% of the current *R. irregularis* EST collection [23] indicating that it represents nearly the complete genic region of the genome. This is further supported by a survey of core eukaryotic genes (CEG), which shows that among the 248 CEG proteins 229 (92.3%) are included in the predicted protein-coding genes (Table S4). Genome annotation using EVidenceModeler resulted in 27,392 protein-coding gene models representing 30,003 putative transcripts. Of these models 11,145 are supported by at least one *R. irregularis* EST, whereas an additional 5,586 protein-coding gene models find support by homology to available protein sequences. Using an AHRD functional annotation pipeline we could assign putative functions to 14,073 protein-coding gene models (Table S5). To obtain insight into the *R. irregularis* gene repertoire a comparative approach using OrthoMCL was conducted on 10 species representing all five fungal phyla (Fig. 3). This resulted in 19,300 putative orthology groups (Table S6), of which 1,370 contained exclusively *R. irregularis* gene models that may represent genes unique for AM fungi (14,742 gene models in total). Of these 6,014 were functionally annotated (Table S7). A summary of the top ten Interpro domains is shown in Table S8. Interestingly, about 28% of these putative genes are predicted to encode proteins with a kinase domain, underling a striking overrepresentation of these signaling proteins in the *R. irregularis* genome. The second largest group (∼25%) that seems to be enriched especially in *R. irregularis* are BTB domain containing proteins (BTB-POZ (PF00651) and BTB-Kelch (BACK; PF07707)). Both findings are supported a recent transcriptome study [23].

We observed a high level of putative/predicted (retro-)transposable (TE) elements in the *R. irregularis* genome. In addition to well-known TE classes, representing 1.1% of the genome based on the Repbase [24] TE library (Table S9), potential novel TE repeats were identified, revealing that TE repeats represented ∼40% of the genome (Table S10). The presence of potential deleterious TE elements is difficult to reconcile with the ancient asexuality of Glomeromycota, as an uncontrolled accumulation of such elements would cause a deleterious load that leads to extinction [25], [26]. Therefore, the presence of such TE elements [27], together with the identification of meiotic recombination proteins [3] and signatures of recombination within populations [28]–[30], argues for the potential rare occurrence of so far unidentified sexual reproduction in *R. irregularis* [25], [26]. As an alternative, parasexual cycles where nuclei fuse and undergo recombination, together with observed exchange of nuclei through anastomoses, may explain both the spread of TE elements as well as restrain their intragenomic proliferation [2], [11].

### Glomeromycota are related to Mucoromycotina

We noted that the gene repertoire of *R. irregularis* overlaps the most with the repertoire of sequenced Mucoromycotina species. Mucoromycotina have traditionally been classified as Zygomycota, which also have coenocytic hyphae, similarly as those in AM fungi. In general they are saprotrophic fungi, but some isolates can also act as opportunistic pathogens. A reconstruction of the early evolution of fungi largely based on the 45S rDNA locus suggested that the Zygomycota phylum is paraphyletic and that Glomeromycota are sister to the Dikarya phyla Ascomycota and Basidiomycota [31]. However, this has only limited statistical support, and analyses based on protein coding genes gave conflicting results [3], [32], [33]. As our data, together with that from others [12], [18] revealed that the 45S rDNA locus of *R. irregularis* is highly polymorphic we reinvestigated the phylogenetic relationships of *R. irregularis* within the fungi. To do so, we analysed a supermatrix of 35 highly conserved, putative single copy nuclear genes proposed by Capella-Gutiérez et al. [34], totaling a concatenated length of 26,604 aligned amino acids from 23 fungal species and 4 outgroups (Table S11). Phylogenetic analysis of this supermatrix using maximum-likelihood (ML) revealed that *R. irregularis* is related to Mucoromycotina rather than to the Dikarya phyla Basidiomycota and Ascomycota (Fig. 3). This phylogenetic placement of *R. irregularis* received maximal bootstrap support (100%; Fig. 3) and alternative placements resulted in significantly lower likelihoods (p< = 0.004; see Table S12). This finding is in concordance with gene repertoire reconstructions presented here, as well as phylogenetic studies based on genes encoding (meiotic) DNA repair proteins [3], [35], [36]. We note, however, that our taxonomic sampling includes Mucorales only. Additional lineages within Mucoromycotina (i.e. Mortierellales, Endogonales) and especially other currently unplaced subphyla traditionally classified as Zygomycota (e.g. Kickxellomycotina, Zoopagomycotina, Entomophthoromycotina) may better resolve the precise relationships of *R. irregularis*, as genome sequences for these members will become available in the future.

### *R. irregularis* has a relatively small repertoire of effector-like proteins

**Figure 4:**
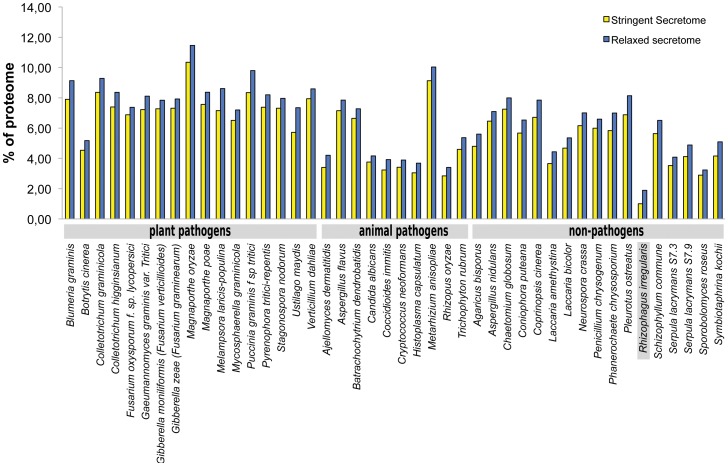
Comparison of secretomes of *R. irregularis* and other 43 fungi. Percentage of predicted proteome representing putative effectors, using stringent (lacking transmembrane domains; yellow bars) or relaxed criteria (including proteins with predicted single transmembrane domain that overlapped with the signal peptide; blue bars).

**Figure 5:**
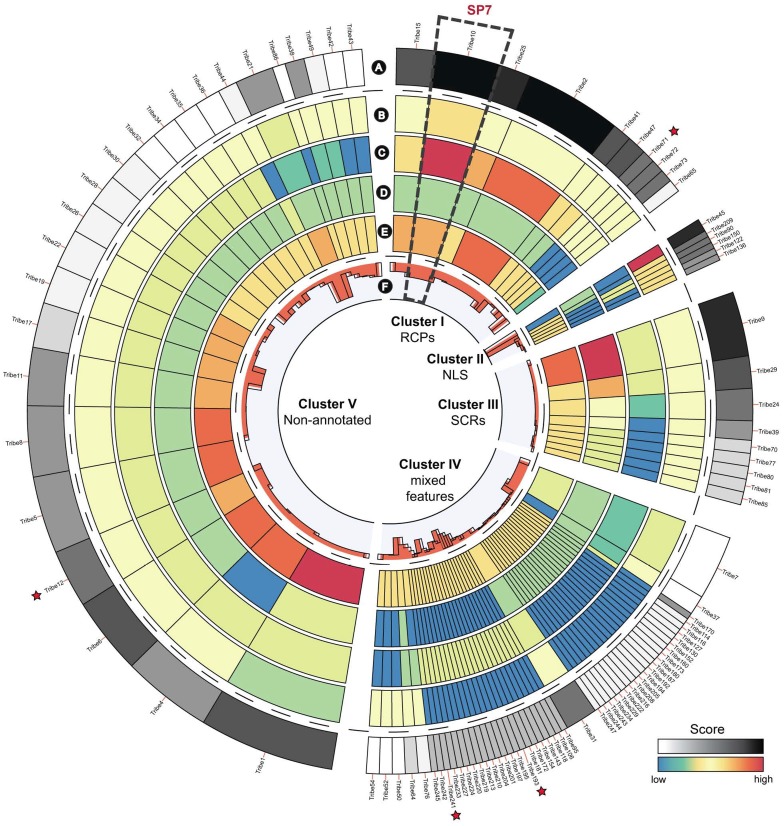
Top 100 ranked protein tribes containing putative effector candidates. Clusters were determined using hierarchical clustering of the top 100 ranked tribes containing putative effector candidates. A. Rank associated with each tribe based on their content of effector features. B. Score for number of members containing a nuclear localization signals (NLS). C. Score for number of members classified as repeat containing (RCPs). D. Score reflecting number of members classified as small and cysteine rich (SCRs). E. Score for number of members not annotated by searches against swissprot. F. Average protein sequence length for tribe members (ranging from 55 to 856 amino acids). Stars indicate tribes that contain members with similarity to the characterised effector SP7.

In comparison to pathogenic fungi, AM fungi have an extremely broad host range. Pathogenic fungi suppress defence responses of their host by secreting effectors that interfere with this defence. This raises the question whether a particular repertoire of secreted putative effector proteins underlies the broad host range of AM fungi. From the deduced proteome of 30,003 putative proteins, we predicted the secretome to contain 299 proteins (1% of proteome) using stringent bioinformatics criteria, and 566 proteins (1.9% of proteome) using more relaxed criteria (Table S13). In relative sense, this is rather low compared with averages of other fungal secretomes such as plant pathogens (7.4%), animal pathogens (4.7%), and non-pathogens (5.3%) (Fig. 4). It is remarkable that AM fungi are able to colonize a broad range of plants despite the fact that it has a small secretome suggesting more research is needed on the effectors. The relative small secretome may have resulted from adaptation to a symbiotic lifestyle in which the secretome has been streamlined through the loss of unnecessary secreted protein genes. The proteins in the *R. irregularis* secretome identified with relaxed criteria were grouped into 254 tribes based on sequence similarity, annotated, and ranked based on potential effector features (Table S13). The top 100 tribes that are likely to contain effectors highlighted five protein tribes containing thirteen sequences with similarity to the known *R. irregularis* effector protein SP7 (Fig. 5) [37]. Alignment of these protein sequences identified conserved features also present in SP7 (Fig. S5), indicating that these proteins are good candidates to display effector functionality. To further analyze potential *R. irregularis* specific features, we compared the number of predicted secreted proteins of *R. irregularis* in each tribe with those of selected pathogenic and symbiotic fungi (Fig. S6). A survey of top 100 tribes, containing 16–134 members, revealed that *R. irregularis* was represented in only 26 tribes compared to for example 76 tribes and 64 tribes for the fungi *Magnaporthe oryzae* and *Laccaria bicolor*, respectively. This suggests that not only the secretome of *R. irregularis* is reduced, but also that it is missing some secreted proteins that are present in other fungi compared in this analysis. However, there is a 22-member tribe composed of *R. irregularis* proteins only (Tribe 62 based on the numbering of Fig. S6, equivalent to the largest *R. irregularis* Tribe 1 of Table S13). It is tempting to speculate that such effectors play important roles in the AM symbiosis.

Among the putative effectors, a protein with a so-called Crinkler (CRN) domain was present (RirT087480; tribe 245). Secreted CRN domain effectors are abundantly present in oomycete plant pathogens of the *Phytophthora* genus [38], [39]. We searched the *R. irregularis* deduced proteome for proteins containing CRN domains using amino acid sequences of canonical CRN proteins from the potato blight pathogen *Phytophthora infestans* as query. This resulted in 42 sequences with positive scores for the so-called N-terminal LFLAK domain that is common to all CRN proteins (Table S14). Within this set, we also identified additional CRN domains (Fig. S7, Table S14). Among these 42 CRN-like proteins, only five have a putative signal peptide, similar as the canonical CRN proteins from *P. infestans*. Similar CRN domain effector-like proteins were identified in the Chytrid fungus *Batrachochytrium dendrobatidis*, but not yet in other sequenced fungal genomes. This led to speculations of horizontal acquisitions of these genes by this pathogenic fungus [40]. However, the occurrence of CRN genes in the *R. irregularis* genome makes a vertical descent equally well possible, and indicates that these proteins are encoded by an ancient eukaryotic gene family.

### Conclusion

Genome sequencing of individual cells has previously been used for example to determine the genome of individual cancer cells [41]. However in these cases a reference genome was already available. Our study shows that it is possible to obtain a *de novo* genome sequence starting from a single haploid nucleus. This approach can be attractive for genomes of species with high heterozygocity that are notoriously difficult to assemble. We applied a single nucleus genome sequence approach on the AM fungus *R. irregularis* and provide solid evidence for the occurrence of homokaryosis in this strain. This demystifies the long lasting hypothesis that nuclei of a single *Rhizophagus* isolate are markedly different. The sequences of four nuclei, in combination with the reference genome sequence will provide the basis for future studies on AM fungi to address issues such as genetic selection, long-term persistence of asexuality, obligate endosymbiosis, adaptation to host plants and suppression of plant defense.

## Materials and Methods

### Isolation of nuclei, DNA extraction and whole genome amplification

A monoxenic culture of *Agrobacterium rhizogenes* (RiT-DNA) transformed chicory (*Cichorium intybus*) roots mycorrhized with the fungus *R. irregularis* DAOM197198 was obtained from Dr. Paola Bonfante and Dr. Andrea Genre (University of Torino) (originally obtained from GINCO (MUCL 43194)). This root culture was designated DAOM197198w and grown in a split-plate setup, where the fungus is allowed to grow into a compartment containing liquid M medium to allow easy collection of spores and extraradical mycelium [42].

Genomic *R. irregularis* DNA, used for meta-genome sequencing, was isolated from extraradical mycelium containing spores using the DNeasy Plant kit (Qiagen). Mycelium containing spores was washed 10× in sterile water. Spores were carefully teased out using forceps, washed by transferring through a series of (at least 5) sterile water droplets, and finally transferred to a small drop of 10 µM Sytox Green (Invitrogen) in Citifluor (Citifluor Ltd). To release the nuclei, spores were crushed using a teflon coated dounce and transferred to an eppendorf tube. The volume was adjusted to 25 µl with 10 µM Sytox Green. To remove cell debris, the crushed spore suspension was centrifuged for 1 min. at 4000 rpm.

Spore suspensions were loaded onto cover slips, from which individual nuclei were collected using a Narishige micromanipulator mounted to an inverted PASCAL Zeiss Confocal Laser Scanning microscope (excitation 480 nm; emission 505–530 nm). Individual isolated nuclei were transferred to a PCR tube containing 5 µl 1× ALB (200 mM KOH, 0.5 mM DTT) buffer, by breaking the tip of the glass microinjection needle containing the captured nucleus.

Whole genome amplification (WGA) was performed using the REPLI-g UltraFast midi-kit (Qiagen) according to the manufacturers instructions. Amplified DNA was diluted 100×. To verify the efficiency of the WGA a set of 10 selected amplicons was amplified using Premix Taq (Ex TaqVersion 2.0) polymerase (Takara Bio Inc). Amplicons could not be amplified from WGA-amplified control suspension lacking single nuclei. The extent of contamination of the WGA amplified DNA with bacterial DNA was checked by amplification of 16S rDNA amplicons. Primers for selected amplicons are listed in Table S15. From in total 40 WGA samples, 4 samples that allowed amplification of the selected *R. irregularis* amplicons and showed minimal bacterial contamination were selected for Illumina sequencing.

### Illumina sequencing and assembly

#### Library construction and sequencing

The amplified DNAs were sheared into fragments of about 350 bp, using an ultrasonicator (Covaris), to construct a paired-end sequencing library for each sample according to the manufacturer's instructions (Illumina). All libraries were paired-end sequenced with a read length of 90 bp for each end on the Illumina Hiseq 2000. The duplicated reads, low-quality and adaptor sequences from each library were removed (Table S1).

#### Assembling for each sample

Paired-end reads from each sample were separately assembled by employing k-mer of optimized length (N31: 59; N33: 63; N36: 59; N6: 60; DNA1: 63; DNA2: 63) using SOAPdenovo2 [43]. Then, all paired-end reads were aligned to the assembled contigs. If two contigs were connected by more than 3 read pairs, they were constructed into a scaffold. Only the scaffolds with the length >100 bp were remained in the final assembly. In addition, the quality of each base was corrected by mapping the reads onto the assembly.

#### Assembling all reads from the six samples

A total of 21.5 Gb raw sequence data representing 150-fold coverage of *R. irregularis* genome were generated for the six samples. To reduce the sequencing errors to a large extent and to facilitate the assembly of the sequencing data from different samples, we also performed error correction using k-mer frequency spectrum. We used the MSR-CA assembler version 1.6 (ftp://ftp.genome.umd.edu/pub/MSR-CA/), which combines the advantage of de Bruijn graph and Overlap-Layout-Consensus assembly approaches, to generate the reference genome assembly. During the assembly, the program will compute the optimal k-mer size based on the read data and GC content (25–101 bp are supported). All contigs with the length of less than 200 bp were excluded in the final assembly.

### Genome and gene functional annotation

#### Masking repeats

The genomic scaffolds were masked using RepeatMasker (http://www.repeatmasker.org, version 3.3.0) and the Repbase TE library [24] for identifying transposable elements across the genome. We found that the percentage of known transposable elements in the genome was about 1.1%.

Three software packages, PILER (version 1.0) [44], LTR_FINDER (version 1.05) [45], and RepeatScout (version 1.0.5) [46], were used to identify *de novo* repetitive elements in the reference genome, which was previously masked with Repbase TE library (version 20120418). Firstly, repetitive elements which belong to rRNA or satellites were filtered using BLASTN with parameters of E-value≤1e-10, identity ≥80%, coverage ≥50%, and match length ≥100 bp. Secondly, if comparison of two identified repeats met the criteria of E-value≤1e-10, identity ≥80%, coverage ≥80%, and match length ≥100 bp, then the shorter one was excluded. Through these two filtering steps, a non-redundant *de novo* transposable elements database was generated. Finally, RepeatMasker (version 3.3.0) was used to re-mask the reference genome with this *de novo* transposable elements' database, and we identified ∼40% transposable elements in the reference genome.

#### Identification of putative variants among a group of samples using Cortex pipeline

Cortex, designed for reference-free variant calling by *de novo* assembly of multiple samples, allows directly comparing samples without using a reference genome [47], [48]. We applied Cortex to data from (1) the four single nucleus samples, (2) the two mycelium samples, respectively. Thus, we could compare both results without a reference. We used the joint discovery workflow to directly compare all samples from the same group by using the Bubble Caller algorithm. In this workflow, we set the reference to be “Absent”, meaning that no reference was loaded into the graph and a fake reference is used to get the coordinates of variants. In addition, as suggested, we set k = 31(low k-mer for relatively low coverage at these sites) and k = 61(high k-mer for genome repeat content/genome complexity) to make different variants accessible.

#### Prediction of protein-coding genes

EVidenceModeler (EVM, version r03062010) [49], which is a nonstochastic weighted evidence evaluation system to produce consensus gene structure, was used to combine the alignments of proteins and transcripts to the genomic sequences, and various *de novo* predictions into a predicted gene set. A more detailed explanation as follows. Firstly, we processed evidence at the transcript level. Spaln (version 1.4.4) [50] mapped the fungal ESTs downloaded from NCBI (Sep 2012) onto our assembled genome and mapping by PASA (version rJAN_09_2011) [51] used *R. irregularis* ESTs [23]. These two processes/programs produced a dataset of putative intron-exon boundaries. Meanwhile, the alignment of ESTs to the reference genome by PASA also produced protein-coding gene models. Based on this set of gene models, we constructed a training set, which was used by *de novo* predictors, by selecting the genes with complete structures and at least 95% mapping rate for UniProt [52] proteins, and filtering out the redundant genes with more than 70% sequence identity by CD-HIT (version 4.1.1) [53]. Secondly, we focused on the evidence at the protein sequence level. The protein sequences from UniProt fungi (release 2012_09) [52] were mapped onto the genomic sequence using Spaln (version 1.4.4) [54] and TBLASTN [55]. The putative intron-exon boundaries were generated by Spaln. For TBLASTN mapping, we performed following procedures: (I) For each protein, joining all of the HSPs (1e-5) with the gap of 500 bp into a consecutive region; (II) selecting the region when the overlapping coverage of its HSPs with the protein is greater or equal to 80%; (III) extending 1000 bp at both ends of the region; (IV) applying GeneWise onto the region to identify the putative intron-exon boundaries of the predicted gene. Thirdly, we collected protein-coding evidence by *de novo* predictors. For this purpose, AUGUSTUS (version 2.4) [56], GeneID (version 1.4.4) [57], GeneMark-ES (version 2.3) [58], GlimmerHMM (version 3.0.1) [59] and SNAP (2006-07-28) [60] were used. Besides GeneMark-ES, all programs used the masked genomic sequences. August, GlilmmerHMM and Snap are supervised predictors with the training set generated by PASA abovementioned, while GeneID utilized the parameters of *Schizosaccharomyces japonicus*. Finally, all evidence for protein-coding genes collected by the methods abovementioned was combined into a consensus protein-coding gene models by EVM. In addition, based this set of gene models and the EST dataset, we also used PASA to polish the gene models by adding untranslated regions (UTRs), correcting gene models, and generating all possible alternatively spliced isoforms at the mRNA level.

#### Functional annotation

The putative biological functions of the protein-coding genes predicted were assigned by AHRD (developed by Schoof et al. https://github.com/groupschoof/AHRD), which integrates three types of evidence to describe gene functions using standard nomenclature. The three types of evidence are: (I) The best BLASTP alignments (E-value cutoff of 1e-4) of the SwissProt database (release 2011-03) [52] and yeast protein sequences downloaded from NCBI (2012-12-10); (II) The InterPro signatures determined by searching against the InterPro databases (v29.0) [61] with InterProScan (V4.7) [62]; (III) The GO terms assigned by BLAST2GO (version 2.5.0) [63] based on the gene ontologies (GO version 2012-11-03).

CEGMA (http://korflab.ucdavis.edu/datasets/cegma/) analysis was performed according to [64], to assess the completeness of the assembly.

### Orthology assessment

OrthoMCL [65] was used to identify orthologous groups among the set of protein sequences extracted from the following eleven completely sequenced genomes: *R. irregularis*, *Neurospora crassa*, *Tuber melanosporum, Saccharomyces cerevisiae, Laccaria bicolor, Ustilago maydis, Rhizopus oryzae, Phycomyces blakesleeanus, Batrachochytrium dendrobatidis, Magnaporthe grisea* and *Monosiga brevicollis* [66]–[75]. Only the longest sequence of each protein-coding gene was chosen in the further analysis. The set contains 171,398 sequences. Three steps took as follows: (1) all-against-all comparison strategy was applied to the set of protein sequences by BLASTP with an E-value cutoff of 1e-5; (2) The distance matrix among all proteins was constructed by the OrthoMCL algorithm; (3) The orthologous groups were generated by MCL [76] (I = 1.5) algorithm based on the distance matrix. The software versions used in this process were: OrthoMCL version 2.02, MCL version mcl 10–201, and NCBI BLAST version 2.2.15.

### Phylogenetic analyses

We reinvestigated the phylogenetic placement of *R. irregularis* within the fungi based on a set of 52 low-copy genes proposed by [34] with addition of orthologs from *R. irregularis*, *Magnaporthe orzyzae*, *Tuber melanosporum*, *Ustilago maydis*, and the Cryptomycete *Rozella allomyces* [77]. Amino acid sequences were aligned using MAFFT [78] and positions covering less than three species were trimmed. Seventeen gene alignments supported paralogy shared among different fungal lineages and were excluded from the analysis, leaving in a total number of 35 gene alignments that were concatenated into a supermatrix of 26,604 amino acids. Table S14 lists all included protein sequences. We then estimated a ML phylogenetic tree based on the supermatrix using RAxML 7.2.8 [79] applying the amino acid substitution model with the best fit on a maximum parsimony tree (rtREV; [80] with empirical frequencies and gamma-distributed rate heterogeneity (-m PROTGAMMARTREVF). Clade support was assessed using the rapid bootstrapping algorithm [81] with 100 alignment replicates.

To test alternative hypotheses of monophyly we imposed three alternative topological constraints on parallel RAxML analyses, with *R. irregularis* forming a clade with either Dikarya, Chytridiomycota, or Microsporidia and Cryptomycota. Branch lengths were optimized and all competing hypotheses were compared with an unconstrained analysis using the eight bootstrap probability tests implemented in CONSEL [82]; Table S12).

### Effector mining

#### Identifying fungal secretomes

Proteomes of 43 fungi containing 17 plant pathogens, 10 animal pathogens and 16 non-pathogens were used to identify the secretomes of the fungi including *R. irregularis*. Therefore, we used the following approach; First, signal peptide containing proteins were predicted using SignalP V2.0 software [83] using the criteria of Torto et al. [84]. Second, the presence of transmembrane domains and mitochondrial signal peptides in these proteins was predicted using TMHMM V2.0c (http://www.cbs.dtu.dk/services/TMHMM/) and TargetP V1.1 [85] programs. Third, secretomes were established by removing the proteins that contain transmembrane domains and mitochondrial signals. For stringent prediction of secretome, proteins with one or more transmembrane domains were removed. For relaxed prediction of secretomes, proteins with single transmembrane domain that overlapped with the signal peptide were included in the secretome. Last, the secretome was assessed for the presence of endoplasmic reticulum (ER) retention signal by either searching for the canonical ER retention signal sequence “KDEL or HDE[LF]” [86] or by using the protein localization prediction program WoLF PSORT [87]. However, we would like to point out that in our experience these ER retention signals are not particularly robust for fungal proteomes.

#### Annotation and classification of candidate effectors

To identify and classify candidate effectors from *R. irregularis*, we implemented a modified version of the bioinformatics pipeline described in Saunders et al. [88]. Briefly, proteins in the secretome were annotated with (I) nuclear localization signal (PredictNLS, [89] and Prosite Scan with database release 20.91, [90], (II) cysteine content higher than 3% [88], [91], (III) repeat units (T-REK, [92], and (IV) BLASTP [93] hit against UniProtKB/Swiss-Prot protein database [94]. The proteins were then grouped into tribes based on sequence similarity of the mature proteins using Markov clustering [78]. To order and classify the secreted protein tribes, we used the aforementioned annotation criteria and associated the scores to each tribe based on their likelihood of containing potential effector proteins. Tribes were then ranked giving a higher weight to features that are distinctive to the only reported *R. irregularis* effector SP7 [37].

#### Identification of CNR-like proteins in *R. irregularis*

To identify CRN-like proteins in *R. irregularis*, we did BLASTP search with amino acid sequences of canonical CRN proteins from *P. infestans* against the *R. irregularis* proteome. We collected sequences that matched to CRN sequences with E-value less than 10^−5^ and searched for CRN motifs using a library of 36 CRN HMMs described in Haas et al [39]. 90 sequences were identified that had similarities to *P. infestans* CRN proteins from BLASTP search with E-value cutoff of 10^−5^. Among these, 42 sequences showed positive scores for LFLAK_domain HMM, which is common to all CRN proteins (Fig. S6, Table S14). Within this set, other CRN domains described in Haas et al. [39] were additionally identified, including DWL (18 proteins with positive score), DI (1), D2 (2), DBF (2), DC (1), DN5 (1), DN17 (10), DSV (1), DX8 (1), DX9 (1), DXS (2), and DXX (5) domains (Fig. S6, Table S14). SignalP2.0 was used to predict signal peptides, with HMM probability scores from 0.508 to 0.971, which are comparable to the canonical CRN proteins from *P. infestans*, which have scores of 0.541 to 0.984 [39]. CRN-domain containing proteins with scores less than 0.9 cutoff used for secretome prediction were omitted from the secretome. Trans-membrane domains were predicted by TM-HMM 2.0c program.

### Accession numbers

The sequence data have been deposited into Genbank with accession number PRJNA230015. The *R. irregularis* reference genome and assemblies are also available at http://cmb.bnu.edu.cn/Rhizophagus_irregularis_v10/.

## Supporting Information

Figure S1 Dot-plot of the reference genome assembly against itself. To rule out potential artificial duplications, the reference assembly was self-aligned, using MUMmer (with default settings). If more than 70% of a scaffold sequence can be aligned to other sequences with identity > = 95%, it is considered as potential artificial duplication. Only 9.0 Mb (6.4%) involving 8,147 scaffolds met these criteria, indicating a largely non-redundant genome assembly.
(PDF)

Figure S2 Alignments of POL1-like (PLS) sequences (RirG174000) across seven *R. irregularis* assemblies. The *PLS* sequence AY330523.1 of Glomus etunicatum was used to identify the homologous sequences in the six assemblies.
(PDF)

Figure S3 Alignments of BIP sequences across the seven *R. irregularis* assemblies. The *BIP* sequence AJ319763.1 was used to identify the homologous sequences in the assemblies.
(PDF)

Figure S4 Alignments of Bg112 sequences across seven *R. irregularis* assemblies. The *Bg112* sequence GU930824.1 was used to identify the homologous sequences in the six assemblies.
(PDF)

Figure S5 Sequence alignment of SP7-like putative effectors reveals conservation around SP7 features. Illustration of the consensus sequence from alignment of thirteen protein sequences with similarity to the characterised effector SP7.
(PDF)

Figure S6 Distribution of *R. irregularis* putative effectors in fungal tribes. Tribes were constructed from secretomes of selected plant-pathogenic and symbiotic fungi using Tribe-MCL as described in Haas et al. [39]. ABIS, *Agaricus bisporus*; BCIN, *Botrytis cinerea*; BGRA, *Blumeria graminis*; FOXG, *Fusarium oxysporum*; LAME, *Laccaria amethystina*; LBIC, *Laccaria bicolor*; MORY, *Magnaporthe oryzae*; MLAR, *Melampsora laricis-populina*; NCU, *Neurospora crassa*; PGRA, *Puccinia graminis* f. sp. *tritici*; RIRR, *Rhizophagus irregularis*; UMAY, *Ustilago madis*; VDAH, *Verticillium dahliae*.
(PDF)

Figure S7 CRN domains identified in *R. irregularis*. The diagram shows the structure of CRN domains in *Phytophthora infestans* (reproduced from Haas et al [39]). Blue stars indicate the domains identified in the 42 *R. irregularis* CRN-like sequences; LFLAK domain (42 proteins with positive score), DWL (18 proteins with positive score), DI (1), D2 (2), DBF (2), DC (1), DN5 (1), DN17 (10), DSV (1), DX8 (1), DX9 (1), DXS (2), and DXX (5) domains (see also Table S14).
(PDF)

Table S1 Summary of the sequenced read data.
(DOCX)

Table S2 Overview of SNPs and INDELs in each sample identified by mapping its reads onto the reference genome.
(DOCX)

Table S3 Overview of SNPs and INDELs among the four single nuclei and the two mycelial samples based on reference-free variant calling by *de novo* assembly using Cortex. SNP_FROM_COMPLEX (INDEL_FROM_COMPLEX)*: SNP (INDEL) called from composite variants consisting of clusters of nearby SNPs, or SNPs and indels, or large deletions with a small insertion at the breakpoint etc, according to the Cortex manual: http://cortexassembler.sourceforge.net/cortex_var_user_manual.pdf. Total length*: the sum of insertion size, deletion size and the number of SNPs, including SNP_FROM_COMPLEX.
(XLSX)

Table S4 Summary of the core eukaryotic genes (CEG) in the assembly.
(DOCX)

Table S5 The functional annotation of protein-coding genes. Group ID = OrthoMCL group identifier number (corresponding to Supplementary table 3), SubgroupID = OrthoMCL subgroup number, GO = identifiers in Gene Ontology database (www.geneontology.org); PfamA = family domain identifiers in Pfam database (pfam.sanger.ac.uk); InterPro = domain identifiers in InterPro database (www.ebi.ac.uk/interpro).
(XLSX)

Table S6 Putative orthology groups. OrthoMCL clustering of genes from 10 species representing all 5 fungal phyla. Rir = *Rhizophagus irregularis* (Glomeromycota);Sac = *Saccharomyces cerevisiae* (Ascomycota); Nec = *Neurospora crassa* (Ascomycota); Tum = *Tuber melanosporum* (Ascomycota); Mag = *Magnaporte grisae* (Ascomycota); Lab = *Laccaria bicolor* (Basidiomycota); Usm = *Ustilago maydis* (Basisdiomycota); Rho = *Rhizopus oryzae* (Zygomycota); Phb = *Phycomyces blakeseanus* (Zygomycota); Bad = *Batrachochytrium dendrobatidis* (Chytridiomycota); Mob = *Monosiga brevicollis* (Choanoflagellida; outgroup). GO = identifiers in Gene Ontology database (www.geneontology.org); InterPro = domain identifiers in InterPro database (www.ebi.ac.uk/interpro); SGD = annotation retrieved from the Saccharomyces Genome Database (www.yeastgenome.org).
(XLSX)

Table S7 List of annotated protein-coding genes unique for *R. irregularis*. GroupID corresponds with orthoMCL clusters in Table S5. GO = identifiers in Gene Ontology database (www.geneontology.org); PfamA = family domain identifiers in Pfam database (pfam.sanger.ac.uk); InterPro = domain identifiers in InterPro database (www.ebi.ac.uk/interpro).
(XLSX)

Table S8 Summary of top ten Interpro domains in the annotated unique *R. irregularis* proteins.
(DOCX)

Table S9 Classification and distribution of repeats based on Repbase TE library.
(DOCX)

Table S10 Summary of predicted TE elements in the *R. irregularis* genome.
(XLSX)

Table S11 Protein sequences of 35 putative single copy genes used for phylogenetic analysis. GroupID numbers correspond with orthoMCL clusters in Table S5.
(XLSX)

Table S12 P-values of competing topological hypotheses calculated using CONSEL. Based on bootstrap probability tests of sitewise likelihood values under the rtREV model calculated with RAxML; unconstrained = analysis without imposing monophyly contraint (*R. irregularis* forms a clade with Mucoroycotina); Dikarya = constrained analysis imposing *R. irregularis* to form a clade together with Dikarya; Chytridiomycota = same but with Chytridiomycota; Microsporidia = same but with Microsporidia and Cryptomycota; au = approximately unbiased test; np = multiscale bootstrap; bp = bootstrap; pp = Bayesian posterior probability; kh = Kishino-Hasegawa test; sh = Shimodaira-Hasegawa test; wkh = weighted Kishino-Hasegawa test; wsh = weighted Shimodaira-Hasegawa test.
(XLSX)

Table S13 Candidate effectors in *R. irregularis*.
(XLSX)

Table S14 *Rhizophagus irregularis* proteins with positive scores for CRN domain HMMs. Domain analysis according to Haas et al. [39], See: Material and Methods and Fig. S7.
(XLSX)

Table S15 Primers used to verify whole genome amplification.
(XLSX)
