# Supplementary figures and images for: Single Nucleus Genome Sequencing Reveals High Similarity among Nuclei of an Endomycorrhizal Fungus
Source: PLoS Genet. 2014 Jan 9;10(1):e1004078. doi: 10.1371/journal.pgen.1004078 (PMC3886924; doi:10.1371/journal.pgen.1004078)

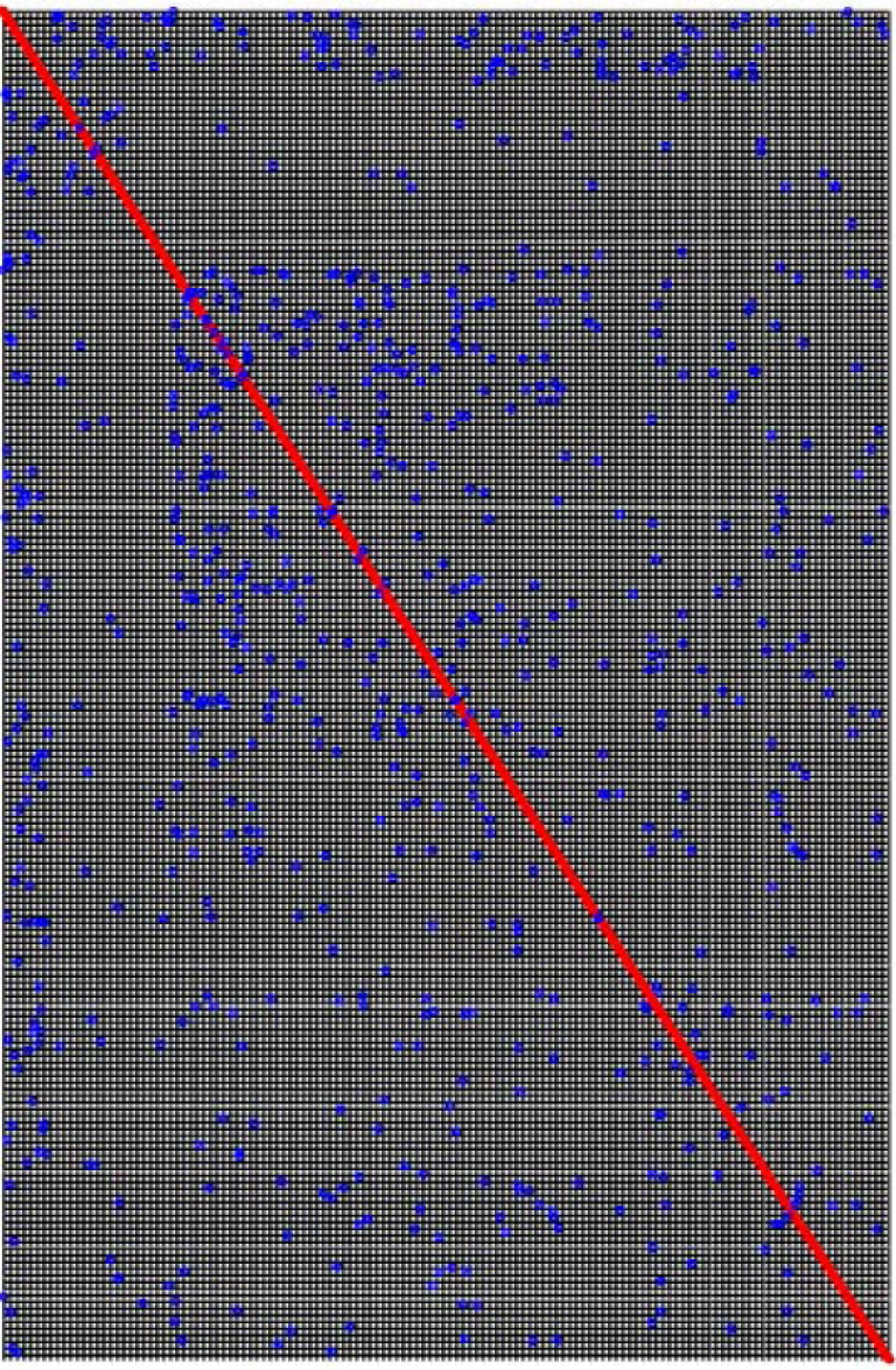

Supplement: Figure S1 — Dot-plot of the reference genome assembly against itself. To rule out potential artificial duplications, the reference assembly was self-aligned, using MUMmer (with default settings). If more than 70% of a scaffold sequence can be aligned to other sequences with identity > = 95%, it is considered as potential artificial duplication. Only 9.0 Mb (6.4%) involving 8,147 scaffolds met these criteria, indicating a largely non-redundant genome assembly. (PDF) [file pgen.1004078.s001.pdf]

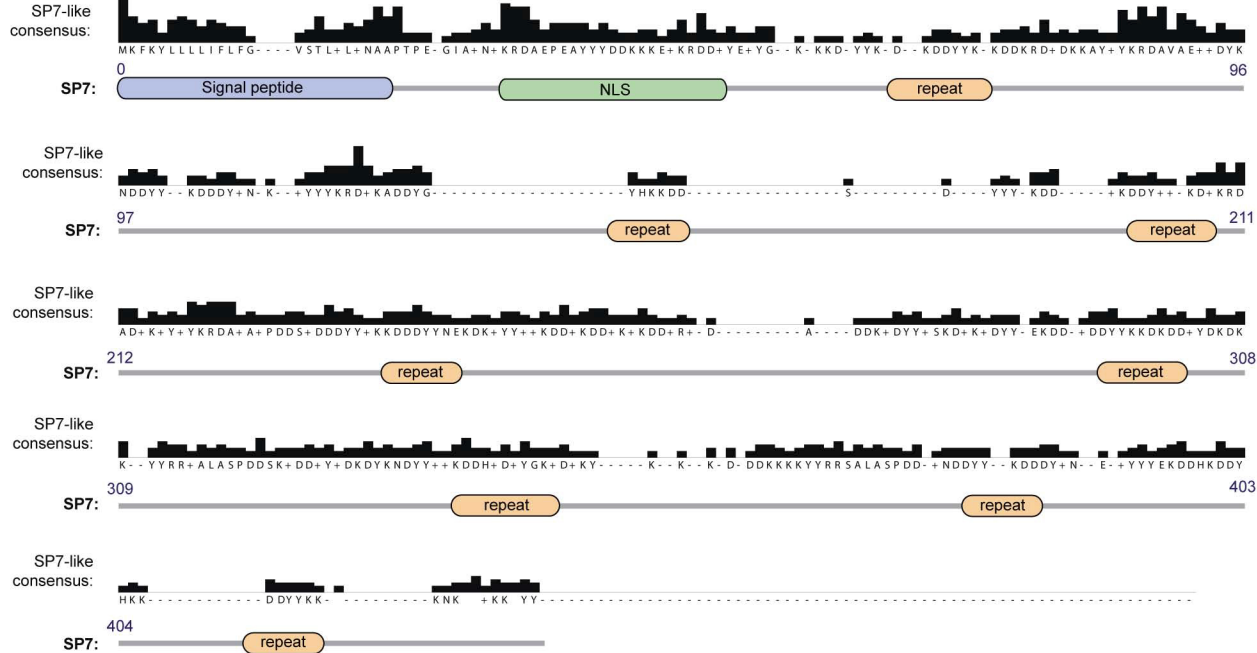

Supplement: Figure S5 — Sequence alignment of SP7-like putative effectors reveals conservation around SP7 features. Illustration of the consensus sequence from alignment of thirteen protein sequences with similarity to the characterised effector SP7. (PDF) [file pgen.1004078.s005.pdf]

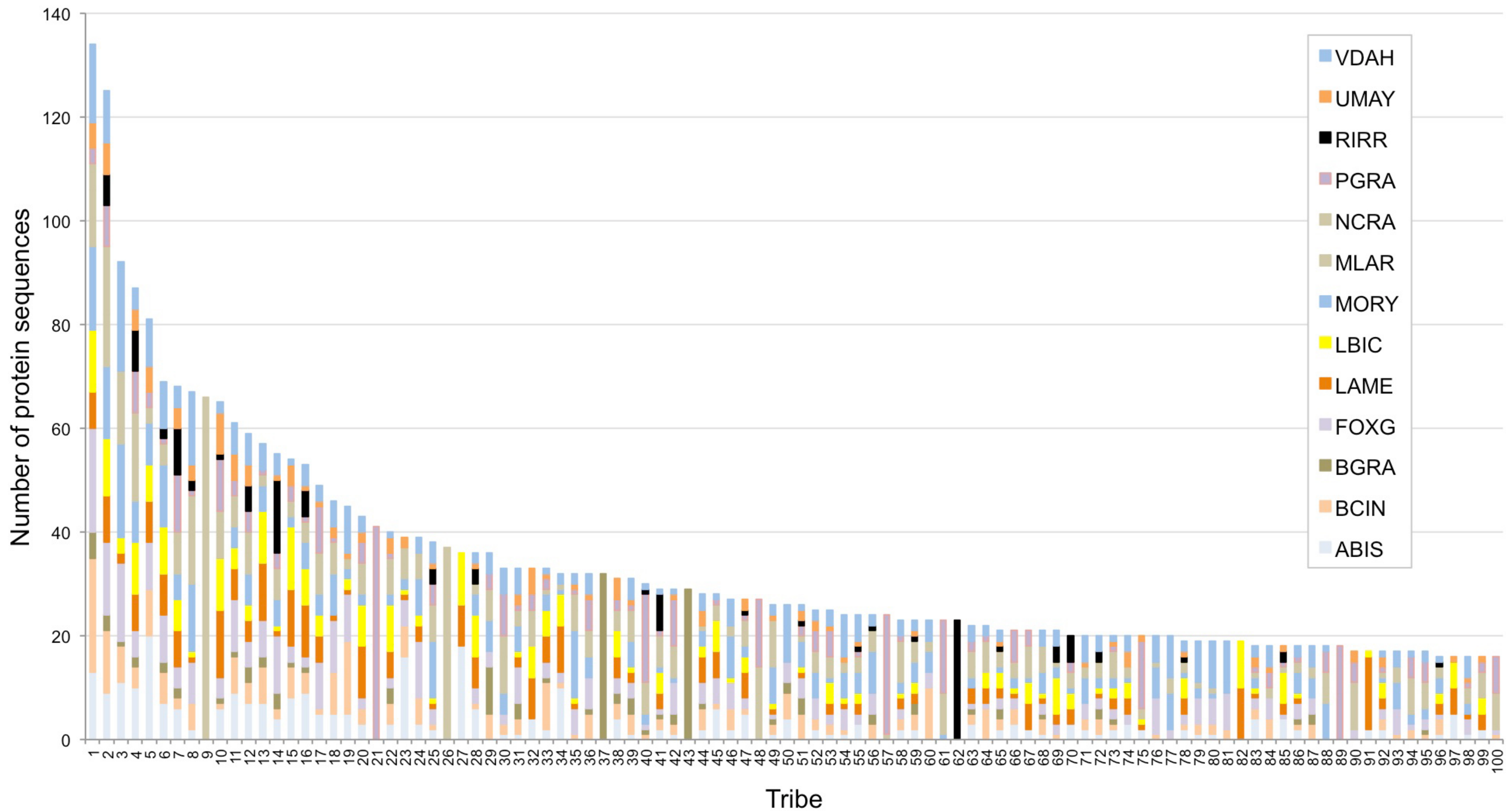

Supplement: Figure S6 — Distribution of R. irregularis putative effectors in fungal tribes. Tribes were constructed from secretomes of selected plant-pathogenic and symbiotic fungi using Tribe-MCL as described in Haas et al. [39]. ABIS, Agaricus bisporus; BCIN, Botrytis cinerea; BGRA, Blumeria graminis; FOXG, Fusarium oxysporum; LAME, Laccaria amethystina; LBIC, Laccaria bicolor; MORY, Magnaporthe oryzae; MLAR, Melampsora laricis-populina; NCU, Neurospora crassa; PGRA, Puccinia graminis f. sp. tritici; RIRR, Rhizophagus irregularis; UMAY, Ustilago madis; VDAH, Verticillium dahliae. (PDF) [file pgen.1004078.s006.pdf]

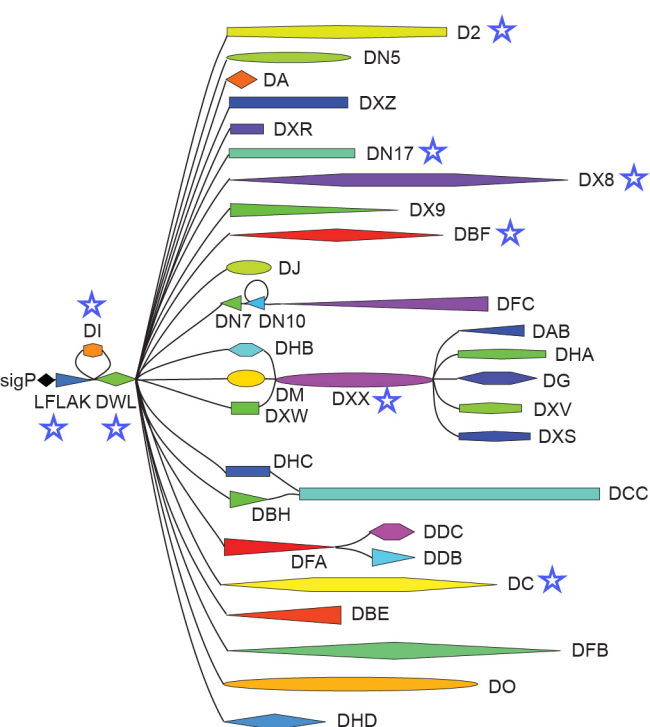

★ = Domains identified in *Rhizophagus irregularis* proteome

Supplement: Figure S7 — CRN domains identified in R. irregularis. The diagram shows the structure of CRN domains in Phytophthora infestans (reproduced from Haas et al [39]). Blue stars indicate the domains identified in the 42 R. irregularis CRN-like sequences; LFLAK domain (42 proteins with positive score), DWL (18 proteins with positive score), DI (1), D2 (2), DBF (2), DC (1), DN5 (1), DN17 (10), DSV (1), DX8 (1), DX9 (1), DXS (2), and DXX (5) domains (see also Table S14). (PDF) [file pgen.1004078.s007.pdf]
