# Supplementary material for: Single Nucleus Genome Sequencing Reveals High Similarity among Nuclei of an Endomycorrhizal Fungus
Source: PLoS Genet. 2014 Jan 9;10(1):e1004078. doi: 10.1371/journal.pgen.1004078 (PMC3886924; doi:10.1371/journal.pgen.1004078)
Supplement: Figure S2 — Alignments of POL1-like (PLS) sequences (RirG174000) across seven R. irregularis assemblies. The PLS sequence AY330523.1 of Glomus etunicatum was used to identify the homologous sequences in the six assemblies. (PDF) [file pgen.1004078.s002.pdf]

```
RirG174000|0|DNA1 ATATTCGTCAAAAGGCTCTTAAACTTACGGCGAATAGTATGTATGGTTGTTTGGGTTATA
RirG174000|0|DNA2 ATATTCGTCAAAAGGCTCTTAAACTTACGGCGAATAGTATGTATGGTTGTTTGGGTTATA
RirG174000|0|Rir  ATATTCGTCAAAAGGCTCTTAAACTTACGGCGAATAGTATGTATGGTTGTTTGGGTTATA
RirG174000|0|N31  ATATTCGTCAAAAGGCTCTTAAACTTACGGCGAATAGTATGTATGGTTGTTTGGGTTATA
RirG174000|0|N33  ATATTCGTCAAAAGGCTCTTAAACTTACGGCGAATAGTATGTATGGTTGTTTGGGTTATA
RirG174000|0|N6   ATATTCGTCAAAAGGCTCTTAAACTTACGGCGAATAGTATGTATGGTTGTTTGGGTTATA
RirG174000|0|N36  ATATTCGTCAAAAGGCTCTTAAACTTACGGCGAATAGTATGTATGGTTGTTTGGGTTATA
                  ************************************************************

RirG174000|0|DNA1 CTTACTCCAGGTTTTATGCCAAGCCGTTAGCTATGTTGATCACTTTCAAAGGTCGTGAAA
RirG174000|0|DNA2 CTTACTCCAGGTTTTATGCCAAGCCGTTAGCTATGTTGATCACTTTCAAAGGTCGTGAAA
RirG174000|0|Rir  CTTACTCCAGGTTTTATGCCAAGCCGTTAGCTATGTTGATCACTTTCAAAGGTCGTGAAA
RirG174000|0|N31  CTTACTCCAGGTTTTATGCCAAGCCGTTAGCTATGTTGATCACTTTCAAAGGTCGTGAAA
RirG174000|0|N33  CTTACTCCAGGTTTTATGCCAAGCCGTTAGCTATGTTGATCACTTTCAAAGGTCGTGAAA
RirG174000|0|N6   CTTACTCCAGGTTTTATGCCAAGCCGTTAGCTATGTTGATCACTTTCAAAGGTCGTGAAA
RirG174000|0|N36  CTTACTCCAGGTTTTATGCCAAGCCGTTAGCTATGTTGATCACTTTCAAAGGTCGTGAAA
                  ************************************************************

RirG174000|0|DNA1 TTCTGGAAAACACTGTTAAATTAGCGGAGAACGAGAAGATGGAGGTATGTTATTTAATAA
RirG174000|0|DNA2 TTCTGGAAAACACTGTTAAATTAGCGGAGAACGAGAAGATGGAGGTATGTTATTTAATAA
RirG174000|0|Rir  TTCTGGAAAACACTGTTAAATTAGCGGAGAACGAGAAGATGGAGGTATGTTATTTAATAA
RirG174000|0|N31  TTCTGGAAAACACTGTTAAATTAGCGGAGAACGAGAAGATGGAGGTATGTTATTTAATAA
RirG174000|0|N33  TTCTGGAAAACACTGTTAAATTAGCGGAGAACGAGAAGATGGAGGTATGTTATTTAATAA
RirG174000|0|N6   TTCTGGAAAACACTGTTAAATTAGCGGAGAACGAGAAGATGGAGGTATGTTATTTAATAA
RirG174000|0|N36  TTCTGGAAAACACTGTTAAATTAGCGGAGAACGAGAAGATGGAGGTATGTTATTTAATAA
                  ************************************************************

RirG174000|0|DNA1 TTTGATTTAATAAGCAAACAAGCGAACGATTCACATTGATTTTTATGCTTTATTTAAGGT
RirG174000|0|DNA2 TTTGATTTAATAAGCAAACAAGCGAACGATTCACATTGATTTTTATGCTTTATTTAAGGT
RirG174000|0|Rir  TTTGATTTAATAAGCAAACAAGCGAACGATTCACATTGATTTTTATGCTTTATTTAAGGT
RirG174000|0|N31  TTTGATTTAATAAGCAAACAAGCGAACGATTCACATTGATTTTTATGCTTTATTTAAGGT
RirG174000|0|N33  TTTGATTTAATAAGCAAACAAGCGAACGATTCACATTGATTTTTATGCTTTATTTAAGGT
RirG174000|0|N6   TTTGATTTAATAAGCAAACAAGCGAACGATTCACATTGATTTTTATGCTTTATTTAAGGT
RirG174000|0|N36  TTTGATTTAATAAGCAAACAAGCGAACGATTCACATTGATTTTTATGCTTTATTTAAGGT
                  ************************************************************

RirG174000|0|DNA1 AATTTATGGAGATACAGATTCTATTATGATTAACACTAAAGCAATAGACATAAATCAAGC
RirG174000|0|DNA2 AATTTATGGAGATACAGATTCTATTATGATTAACACTAAAGCAATAGACATAAATCAAGC
RirG174000|0|Rir  AATTTATGGAGATACAGATTCTATTATGATTAACACTAAAGCAATAGACATAAATCAAGC
RirG174000|0|N31  AATTTATGGAGATACAGATTCTATTATGATTAACACTAAAGCAATAGACATAAATCAAGC
RirG174000|0|N33  AATTTATGGAGATACAGATTCTATTATGATTAACACTAAAGCAATAGACATAAATCAAGC
RirG174000|0|N6   AATTTATGGAGATACAGATTCTATTATGATTAACACTAAAGCAATAGACATAAATCAAGC
RirG174000|0|N36  AATTTATGGAGATACAGATTCTATTATGATTAACACTAAAGCAATAGACATAAATCAAGC
                  ************************************************************

RirG174000|0|DNA1 GCTTGAAGTTGGTAACATCTTAAAAAAGAAAGTCAATGAACGATACAAATTGCTCGAGCT
RirG174000|0|DNA2 GCTTGAAGTTGGTAACATCTTAAAAAAGAAAGTCAATGAACGATACAAATTGCTCGAGCT
RirG174000|0|Rir  GCTTGAAGTTGGTAACATCTTAAAAAAGAAAGTCAATGAACGATACAAATTGCTCGAGCT
RirG174000|0|N31  GCTTGAAGTTGGTAACATCTTAAAAAAGAAAGTCAATGAACGATACAAATTGCTCGAGCT
RirG174000|0|N33  GCTTGAAGTTGGTAACATCTTAAAAAAGAAAGTCAATGAACGATACAAATTGCTCGAGCT
RirG174000|0|N6   GCTTGAAGTTGGTAACATCTTAAAAAAGAAAGTCAATGAACGATACAAATTGCTCGAGCT
RirG174000|0|N36  GCTTGAAGTTGGTAACATCTTAAAAAAGAAAGTCAATGAACGATACAAATTGCTCGAGCT
                  ************************************************************

RirG174000|0|DNA1 TG
RirG174000|0|DNA2 TG
RirG174000|0|Rir  TG
RirG174000|0|N31  TG
RirG174000|0|N33  TG
RirG174000|0|N6   TG
RirG174000|0|N36  TG
                  **
```

Figure S2. **Alignments of POL1-like (PLS) sequences (RirG174000) across seven *R. irregularis* assemblies.** The *PLS* sequence AY330523.1 of *Glomus etunicatum* was used to identify the homologous sequences in the six assemblies.
