# Supplementary material for: Single Nucleus Genome Sequencing Reveals High Similarity among Nuclei of an Endomycorrhizal Fungus
Source: PLoS Genet. 2014 Jan 9;10(1):e1004078. doi: 10.1371/journal.pgen.1004078 (PMC3886924; doi:10.1371/journal.pgen.1004078)
Supplement: Figure S3 — Alignments of BIP sequences across the seven R. irregularis assemblies. The BIP sequence AJ319763.1 was used to identify the homologous sequences in the assemblies. (PDF) [file pgen.1004078.s003.pdf]

BIP1:

```
BIP1_RirG196040|1|DNA1        ATGAAGAAAGCTACTTTCTTTAAATTTATTGCAGTGTTATGTTCACTGTT
BIP1_RirG196040|3|N33         --------------------------------------------------
RiBIP1_RirG196040             ATGAAGAAAGCTACTTTCTTTAAATTTATTGCAGTGTTATGTTCACTGTT
BIP1_RirG196040|0|DNA2        ATGAAGAAAGCTACTTTCTTTAAATTTATTGCAGTGTTATGTTCACTGTT
BIP1_RirG196040|2|N6          ATGAAGAAAGCTACTTTCTTTAAATTTATTGCAGTGTTATGTTCACTGTT

BIP1_RirG196040|1|DNA1        AATAACTATTCCTAATGTAATAGTAATTTCTGATGAGAAACCTAACGTTG
BIP1_RirG196040|3|N33         --------------------------------------------------
RiBIP1_RirG196040             AATAACTATTCCTAATGTAATAGTAATTTCTGATGAGAAACCTAACGTTG
BIP1_RirG196040|0|DNA2        AATAACTATTCCTAATGTAATAGTAATTTCTGATGAGAAACCTAACGTTG
BIP1_RirG196040|2|N6          AATAACTATTCCTAATGTAATAGTAATTTCTGATGAGAAACCTAACGTTG

BIP1_RirG196040|1|DNA1        GAACAGGTATAAAAATTAATTGCTAAATATTGTTCTTTCGAAACTCATAA
BIP1_RirG196040|3|N33         --------------------------------------------------
RiBIP1_RirG196040             GAACAGGTATAAAAATTAATTGCTAAATATTGTTCTTTCGAAACTCATAA
BIP1_RirG196040|0|DNA2        GAACAGGTATAAAAATTAATTGCTAAATATTGTTCTTTCGAAACTCATAA
BIP1_RirG196040|2|N6          GAACAGGTATAAAAATTAATTGCTAAATATTGTTCTTTCGAAACTCATAA

BIP1_RirG196040|1|DNA1        ATTTTAATATTATATTTCATGTATCGTCAGTTATCGGTATAGATTTAGGA
BIP1_RirG196040|3|N33         --------------------------------------------------
RiBIP1_RirG196040             ATTTTAATATTATATTTCATGTATCGTCAGTTATCGGTATAGATTTAGGA
BIP1_RirG196040|0|DNA2        ATTTTAATATTATATTTCATGTATCGTCAGTTATCGGTATAGATTTAGGA
BIP1_RirG196040|2|N6          ATTTTAATATTATATTTCATGTATCGTCAGTTATCGGTATAGATTTAGGA

BIP1_RirG196040|1|DNA1        ACAACTTATTCTTGTGTTGGAGTATACGTAAACGGAAGAGTTGAGATATT
BIP1_RirG196040|3|N33         --------------------------------------------------
RiBIP1_RirG196040             ACAACTTATTCTTGTGTTGGAGTATACGTAAACGGAAGAGTTGAGATATT
BIP1_RirG196040|0|DNA2        ACAACTTATTCTTGTGTTGGAGTATACGTAAACGGAAGAGTTGAGATATT
BIP1_RirG196040|2|N6          ACAACTTATTCTTGTGTTGGAGTATACGTAAACGGAAGAGTTGAGATATT

BIP1_RirG196040|1|DNA1        AACCAATGACCAGGGTAACCGTATCACGCCTTCTTATGTTGCCTTCACTG
BIP1_RirG196040|3|N33         --------------------------------------------------
RiBIP1_RirG196040             AACCAATGACCAGGGTAACCGTATCACGCCTTCTTATGTTGCCTTCACTG
BIP1_RirG196040|0|DNA2        AACCAATGACCAGGGTAACCGTATCACGCCTTCTTATGTTGCCTTCACTG
BIP1_RirG196040|2|N6          AACCAATGACCAGGGTAACCGTATCACGCCTTCTTATGTTGCCTTCACTG

BIP1_RirG196040|1|DNA1        ATGATGAACGTTTAATTGGTGATGCGGCAAAGAATCAATATTCAAATAAC
BIP1_RirG196040|3|N33         --------------------------------------------------
RiBIP1_RirG196040             ATGATGAACGTTTAATTGGTGATGCGGCAAAGAATCAATATTCAAATAAC
BIP1_RirG196040|0|DNA2        ATGATGAACGTTTAATTGGTGATGCGGCAAAGAATCAATATTCAAATAAC
BIP1_RirG196040|2|N6          ATGATGAACGTTTAATTGGTGATGCGGCAAAGAATCAATATTCAAATAAC

BIP1_RirG196040|1|DNA1        CCCCAGAACACCATTTTTGATTCTAAGCGACTAATTGGTCGTCGTTTTAG
BIP1_RirG196040|3|N33         --------------------------------------------------
RiBIP1_RirG196040             CCCCAGAACACCATTTTTGATTCTAAGCGACTAATTGGTCGTCGTTTTAG
BIP1_RirG196040|0|DNA2        CCCCAGAACACCATTTTTGATTCTAAGCGACTAATTGGTCGTCGTTTTAG
BIP1_RirG196040|2|N6          CCCCAGAACACCATTTTTGATTCTAAGCGACTAATTGGTCGTCGTTTTAG

BIP1_RirG196040|1|DNA1        CGATAAAGAAGTTCAACAAGATATTAAGCACTTTCCATTCAAGGTCATTG
BIP1_RirG196040|3|N33         --------------------------------------------------
RiBIP1_RirG196040             CGATAAAGAAGTTCAACAAGATATTAAGCACTTTCCATTCAAGGTCATTG
BIP1_RirG196040|0|DNA2        CGATAAAGAAGTTCAACAAGATATTAAGCACTTTCCATTCAAGGTCATTG
BIP1_RirG196040|2|N6          CGATAAAGAAGTTCAACAAGATATTAAGCACTTTCCATTCAAGGTCATTG

BIP1_RirG196040|1|DNA1        ACAAAGATGGTAAACCTGTTATTCAAGTTACTGTAAAAGGTGAAGAAAGA
BIP1_RirG196040|3|N33         --------------------------------------------------
RiBIP1_RirG196040             ACAAAGATGGTAAACCTGTTATTCAAGTTACTGTAAAAGGTGAAGAAAGA
BIP1_RirG196040|0|DNA2        ACAAAGATGGTAAACCTGTTATTCAAGTTACTGTAAAAGGTGAAGAAAGA
BIP1_RirG196040|2|N6          ACAAAGATGGTAAACCTGTTATTCAAGTTACTGTAAAAGGTGAAGAAAGA

BIP1_RirG196040|1|DNA1        ATATTTACTCCTGAAGAAATTTCGGCTATGATTCTTGGTAAAATGAAAGA
BIP1_RirG196040|3|N33         --------------------------------------------------
RiBIP1_RirG196040             ATATTTACTCCTGAAGAAATTTCGGCTATGATTCTTGGTAAAATGAAAGA
BIP1_RirG196040|0|DNA2        ATATTTACTCCTGAAGAAATTTCGGCTATGATTCTTGGTAAAATGAAAGA
BIP1_RirG196040|2|N6          ATATTTACTCCTGAAGAAATTTCGGCTATGATTCTTGGTAAAATGAAAGA

BIP1_RirG196040|1|DNA1        AATTGCCGAATCATATTTAGGCAAGAATGTTACACATGCAGTAGTTACAG
BIP1_RirG196040|3|N33         --------------------------------------------------
RiBIP1_RirG196040             AATTGCCGAATCATATTTAGGCAAGAATGTTACACATGCAGTAGTTACAG
BIP1_RirG196040|0|DNA2        AATTGCCGAATCATATTTAGGCAAGAATGTTACACATGCAGTAGTTACAG
BIP1_RirG196040|2|N6          AATTGCCGAATCATATTTAGGCAAGAATGTTACACATGCAGTAGTTACAG

BIP1_RirG196040|1|DNA1        TCCCAGCTTACTTTAATGACGCTCAAAGACAAGCCACAAAAGATGCTGGC
BIP1_RirG196040|3|N33         -----------------GACGCTCAAAGACAAGCCACAAAAGATGCTGGC
RiBIP1_RirG196040             TCCCAGCTTACTTTAATGACGCTCAAAGACAAGCCACAAAAGATGCTGGC
```

```
BIP1_RirG196040|0|DNA2        TCCCAGCTTACTTTAATGACGCTCAAAGACAAGCCACAAAAGATGCTGGC
BIP1_RirG196040|2|N6          TCCCAGCTTACTTTAATGACGCTCAAAGACAAGCCACAAAAGATGCTGGC
                                               *********************************

BIP1_RirG196040|1|DNA1        GTAATTGCTGGACTTAATGTCCTCCGTATTGTAAATGAACCTACGGCGGC
BIP1_RirG196040|3|N33         GTAATTGCTGGACTTAATGTCCTCCGTATTGTAAATGAACCTACGGCGGC
RiBIP1_RirG196040             GTAATTGCTGGACTTAATGTCCTCCGTATTGTAAATGAACCTACGGCGGC
BIP1_RirG196040|0|DNA2        GTAATTGCTGGACTTAATGTCCTCCGTATTGTAAATGAACCTACGGCGGC
BIP1_RirG196040|2|N6          GTAATTGCTGGACTTAATGTCCTCCGTATTGTAAATGAACCTACGGCGGC
                              **************************************************

BIP1_RirG196040|1|DNA1        TGCAATTGCATATGATCTTGATAAATCCGAGGGGGAACGTCAGATTCTTG
BIP1_RirG196040|3|N33         TGCAATTGCATATGATCTTGATAAATCCGAGGGGGAACGTCAGATTCTTG
RiBIP1_RirG196040             TGCAATTGCATATGATCTTGATAAATCCGAGGGGGAACGTCAGATTCTTG
BIP1_RirG196040|0|DNA2        TGCAATTGCATATGATCTTGATAAATCCGAGGGGGAACGTCAGATTCTTG
BIP1_RirG196040|2|N6          TGCAATTGCATATGATCTTGATAAATCCGAGGGGGAACGTCAGATTCTTG
                              **************************************************

BIP1_RirG196040|1|DNA1        TCTATGATCTTGGTGGGGGTACTTTTGATGTGTCTCTCTTGTCAGTCGAA
BIP1_RirG196040|3|N33         TCTATGATCTTGGTGGGGGTACTTTTGATGTGTCTCTCTTGTCAGTCGAA
RiBIP1_RirG196040             TCTATGATCTTGGTGGGGGTACTTTTGATGTGTCTCTCTTGTCAGTCGAA
BIP1_RirG196040|0|DNA2        TCTATGATCTTGGTGGGGGTACTTTTGATGTGTCTCTCTTGTCAGTCGAA
BIP1_RirG196040|2|N6          TCTATGATCTTGGTGGGGGTACTTTTGATGTGTCTCTCTTGTCAGTCGAA
                              **************************************************

BIP1_RirG196040|1|DNA1        GATGATGTTTTTGAGGTATTGGCAACTGCCGGCGATACACATTTGGGAGG
BIP1_RirG196040|3|N33         GATGATGTTTTTGAGGTATTGGCAACTGCCGGCGATACACATTTGGGAGG
RiBIP1_RirG196040             GATGATGTTTTTGAGGTATTGGCAACTGCCGGCGATACACATTTGGGAGG
BIP1_RirG196040|0|DNA2        GATGATGTTTTTGAGGTATTGGCAACTGCCGGCGATACACATTTGGGAGG
BIP1_RirG196040|2|N6          GATGATGTTTTTGAGGTATTGGCAACTGCCGGCGATACACATTTGGGAGG
                              **************************************************

BIP1_RirG196040|1|DNA1        CGAAGACTTCGACAACCGAGTTATTGACCACTTCGTTAAACTTTATAAAA
BIP1_RirG196040|3|N33         CGAAGACTTCGACAACCGAGTTATTGACCACTTCGTTAAACTTTATAAAA
RiBIP1_RirG196040             CGAAGACTTCGACAACCGAGTTATTGACCACTTCGTTAAACTTTATAAAA
BIP1_RirG196040|0|DNA2        CGAAGACTTCGACAACCGAGTTATTGACCACTTCGTTAAACTTTATAAAA
BIP1_RirG196040|2|N6          CGAAGACTTCGACAACCGAGTTATTGACCACTTCGTTAAACTTTATAAAA
                              **************************************************

BIP1_RirG196040|1|DNA1        AGAAAAATAAAATAGATGTTACACAAGATCTAAAAGCTATGGGTAAATTA
BIP1_RirG196040|3|N33         AGAAAAATAAAATAGATGTTACACAAGATCTAAAAGCTATGGGTAAATTA
RiBIP1_RirG196040             AGAAAAATAAAATAGATGTTACACAAGATCTAAAAGCTATGGGTAAATTA
BIP1_RirG196040|0|DNA2        AGAAAAATAAAATAGATGTTACACAAGATCTAAAAGCTATGGGTAAATTA
BIP1_RirG196040|2|N6          AGAAAAATAAAATAGATGTTACACAAGATCTAAAAGCTATGGGTAAATTA
                              **************************************************

BIP1_RirG196040|1|DNA1        AAGCGTGAAGTTGAAAAGGTCAAGCGTACATTATCTTCTCAAATGTCAAC
BIP1_RirG196040|3|N33         AAGCGTGAAGTTGAAAAGGTCAAGCGTACATTATCTTCTCAAATGTCAAC
RiBIP1_RirG196040             AAGCGTGAAGTTGAAAAGGTCAAGCGTACATTATCTTCTCAAATGTCAAC
BIP1_RirG196040|0|DNA2        AAGCGTGAAGTTGAAAAGGTCAAGCGTACATTATCTTCTCAAATGTCAAC
BIP1_RirG196040|2|N6          AAGCGTGAAGTTGAAAAGGTCAAGCGTACATTATCTTCTCAAATGTCAAC
                              **************************************************

BIP1_RirG196040|1|DNA1        TCGTGTCGAAATTGAATCATTTTATGATGGTAAAGATTTTTCCGAAACTT
BIP1_RirG196040|3|N33         TCGTGTCGAAATTGAATCATTTTATGATGGTAAAGATTTTTCCGAAACTT
RiBIP1_RirG196040             TCGTGTCGAAATTGAATCATTTTATGATGGTAAAGATTTTTCCGAAACTT
BIP1_RirG196040|0|DNA2        TCGTGTCGAAATTGAATCATTTTATGATGGTAAAGATTTTTCCGAAACTT
BIP1_RirG196040|2|N6          TCGTGTCGAAATTGAATCATTTTATGATGGTAAAGATTTTTCCGAAACTT
                              **************************************************

BIP1_RirG196040|1|DNA1        TGACCCGAGCCAAATTTGAAGAGCTTAATAATGATCTCTTCCGTAAAACA
BIP1_RirG196040|3|N33         TGACCCGAGCCAAATTTGAAGAGCTTAATAATGATCTCTTCCGTAAAACA
RiBIP1_RirG196040             TGACCCGAGCCAAATTTGAAGAGCTTAATAATGATCTCTTCCGTAAAACA
BIP1_RirG196040|0|DNA2        TGACCCGAGCCAAATTTGAAGAGCTTAATAATGATCTCTTCCGTAAAACA
BIP1_RirG196040|2|N6          TGACCCGAGCCAAATTTGAAGAGCTTAATAATGATCTCTTCCGTAAAACA
                              **************************************************

BIP1_RirG196040|1|DNA1        TTAAAGCCCGTTGAACAAGTGTTGAAAGATGCAAATATTGATAAGAAGGA
BIP1_RirG196040|3|N33         TTAAAGCCCGTTGAACAAGTGTTGAAAGATGCAAATATTGATAAGAAGGA
RiBIP1_RirG196040             TTAAAGCCCGTTGAACAAGTGTTGAAAGATGCAAATATTGATAAGAAGGA
BIP1_RirG196040|0|DNA2        TTAAAGCCCGTTGAACAAGTGTTGAAAGATGCAAATATTGATAAGAAGGA
BIP1_RirG196040|2|N6          TTAAAGCCCGTTGAACAAGTGTTGAAAGATGCAAATATTGATAAGAAGGA
                              **************************************************

BIP1_RirG196040|1|DNA1        TGTACACGATATTGTACTCATTGGTGGTTCCACACGTATTCCCAAAGTTC
BIP1_RirG196040|3|N33         TGTACACGATATTGTACTCATTGGTGGTTCCACACGTATTCCCAAAGTTC
```

```
RiBIP1_RirG196040            TGTACACGATATTGTACTCATTGGTGGTTCCACACGTATTCCCAAAGTTC
BIP1_RirG196040|0|DNA2       TGTACACGATATTGTACTCATTGGTGGTTCCACACGTATTCCCAAAGTTC
BIP1_RirG196040|2|N6         TGTACACGATATTGTACTCATTGGTGGTTCCACACGTATTCCCAAAGTTC
                             **************************************************

BIP1_RirG196040|1|DNA1       AACAACTACTTGAAGAATTCTTTAATGGCAAGAAAGCTTCCAAAAATATT
BIP1_RirG196040|3|N33        AACAACTACTTGAAGAATTCTTTAATGGCAAGAAAGCTTCCAAAAATATT
RiBIP1_RirG196040            AACAACTACTTGAAGAATTCTTTAATGGCAAGAAAGCTTCCAAAAATATT
BIP1_RirG196040|0|DNA2       AACAACTACTTGAAGAATTCTTTAATGGCAAGAAAGCTTCCAAAAATATT
BIP1_RirG196040|2|N6         AACAACTACTTGAAGAATTCTTTAATGGCAAGAAAGCTTCCAAAAATATT
                             **************************************************

BIP1_RirG196040|1|DNA1       AATCCTGATGAAGCGGTTGCGCATGGTGCCGCCATACAAGGAGGTATACT
BIP1_RirG196040|3|N33        AATCCTGATGAAGCGGTTGCGCATGGTGCCGCCATACAAGGAGGTATACT
RiBIP1_RirG196040            AATCCTGATGAAGCGGTTGCGCATGGTGCCGCCATACAAGGAGGTATACT
BIP1_RirG196040|0|DNA2       AATCCTGATGAAGCGGTTGCGCATGGTGCCGCCATACAAGGAGGTATACT
BIP1_RirG196040|2|N6         AATCCTGATGAAGCGGTTGCGCATGGTGCCGCCATACAAGGAGGTATACT
                             **************************************************

BIP1_RirG196040|1|DNA1       TTCCGGAACTGCGAAAAAAAAACTTTTAATAGACGTTTGTCCTCTTACAC
BIP1_RirG196040|3|N33        TTCCGGAACTGCGAAAAAAAAACTTTTAATAGACGTTTGTCCTCTTACAC
RiBIP1_RirG196040            TTCCGGAACTGCGAAAAAAAAACTTTTAATAGACGTTTGTCCTCTTACAC
BIP1_RirG196040|0|DNA2       TTCCGGAACTGCGAAAAAAAAACTTTTAATAGACGTTTGTCCTCTTACAC
BIP1_RirG196040|2|N6         TTCCGGAACTGCGAAAAAAAAACTTTTAATAGACGTTTGTCCTCTTACAC
                             **************************************************

BIP1_RirG196040|1|DNA1       TCGGTATTGAAACAACTGGTGGCGTTATGACAAAATTAATTCCTCGTAAC
BIP1_RirG196040|3|N33        TCGGTATTGAAACAACTGGTGGCGTTATGACAAAATTAATTCCTCGTAAC
RiBIP1_RirG196040            TCGGTATTGAAACAACTGGTGGCGTTATGACAAAATTAATTCCTCGTAAC
BIP1_RirG196040|0|DNA2       TCGGTATTGAAACAACTGGTGGCGTTATGACAAAATTAATTCCTCGTAAC
BIP1_RirG196040|2|N6         TCGGTATTGAAACAACTGGTGGCGTTATGACAAAATTAATTCCTCGTAAC
                             **************************************************

BIP1_RirG196040|1|DNA1       ACTGTAATCCCTACTAAGAAGTCACAAATCTTCTCTACCGCCGCGGATAA
BIP1_RirG196040|3|N33        ACTGTAATCCCTACTAAGAAGTCACAAATCTTCTCTACCGCCGCGGATAA
RiBIP1_RirG196040            ACTGTAATCCCTACTAAGAAGTCACAAATCTTCTCTACCGCCGCGGATAA
BIP1_RirG196040|0|DNA2       ACTGTAATCCCTACTAAGAAGTCACAAATCTTCTCTACCGCCGCGGATAA
BIP1_RirG196040|2|N6         ACTGTAATCCCTACTAAGAAGTCACAAATCTTCTCTACCGCCGCGGATAA
                             **************************************************

BIP1_RirG196040|1|DNA1       CCAACCTACTGTATTAATTCAAGTTTACGAAGGAGAACGTTTTATGACTA
BIP1_RirG196040|3|N33        CCAACCTACTGTATTAATTCAAGTTTACGAAGGAGAACGTTTTATGACTA
RiBIP1_RirG196040            CCAACCTACTGTATTAATTCAAGTTTACGAAGGAGAACGTTTTATGACTA
BIP1_RirG196040|0|DNA2       CCAACCTACTGTATTAATTCAAGTTTACGAAGGAGAACGTTTTATGACTA
BIP1_RirG196040|2|N6         CCAACCTACTGTATTAATTCAAGTTTACGAAGGAGAACGTTTTATGACTA
                             **************************************************

BIP1_RirG196040|1|DNA1       AAGAAAACAACTTGCTCGGCAAGTTCGAATTAACAGGTATACCGCCTGCA
BIP1_RirG196040|3|N33        AAGAAAACAACTTGCTCGGCAAGTTCGAATTAACAGGTATACCGCCTGCA
RiBIP1_RirG196040            AAGAAAACAACTTGCTCGGCAAGTTCGAATTAACAGGTATACCGCCTGCA
BIP1_RirG196040|0|DNA2       AAGAAAACAACTTGCTCGGCAAGTTCGAATTAACAGGTATACCGCCTGCA
BIP1_RirG196040|2|N6         AAGAAAACAACTTGCTCGGCAAGTTCGAATTAACAGGTATACCGCCTGCA
                             **************************************************

BIP1_RirG196040|1|DNA1       CCACGAGGTATACCACAGATCGAGGTTACATTTGAAATTGATACCAATGG
BIP1_RirG196040|3|N33        CCACGAGGTATACCACAGATCGAGGTTACATTTGAAATTGATACCAATGG
RiBIP1_RirG196040            CCACGAGGTATACCACAGATCGAGGTTACATTTGAAATTGATACCAATGG
BIP1_RirG196040|0|DNA2       CCACGAGGTATACCACAGATCGAGGTTACATTTGAAATTGATACCAATGG
BIP1_RirG196040|2|N6         CCACGAGGTATACCACAGATCGAGGTTACATTTGAAATTGATACCAATGG
                             **************************************************

BIP1_RirG196040|1|DNA1       TATAGTGAAAGTATCTGCAGCAGATAAAAGTACAGGGAAAACTGAATCGA
BIP1_RirG196040|3|N33        TATAGTGAAAGTATCTGCAGCAGATAAAAGTACAGGGAAAACTGAATCGA
RiBIP1_RirG196040            TATAGTGAAAGTATCTGCAGCAGATAAAAGTACAGGGAAAACTGAATCGA
BIP1_RirG196040|0|DNA2       TATAGTGAAAGTATCTGCAGCAGATAAAAGTACAGGGAAAACTGAATCGA
BIP1_RirG196040|2|N6         TATAGTGAAAGTATCTGCAGCAGATAAAAGTACAGGGAAAACTGAATCGA
                             **************************************************

BIP1_RirG196040|1|DNA1       TCACAATCATAAACGATAAAGGTCGTCTTTCTCAAGAAGAAATTAATCGT
BIP1_RirG196040|3|N33        TCACAATCATAAACGATAAAGGTCGTCTTTCTCAAGAAGAAATTAATCGT
RiBIP1_RirG196040            TCACAATCATAAACGATAAAGGTCGTCTTTCTCAAGAAGAAATTAATCGT
BIP1_RirG196040|0|DNA2       TCACAATCATAAACGATAAAGGTCGTCTTTCTCAAGAAGAAATTAATCGT
BIP1_RirG196040|2|N6         TCACAATCATAAACGATAAAGGTCGTCTTTCTCAAGAAGAAATTAATCGT
                             **************************************************
```

```
BIP1_RirG196040|1|DNA1        ATGGTTGAAGAAGCCGAACAATTTGCTGAAGAGGATAAGATTCAAAAGGA
BIP1_RirG196040|3|N33         ATGGTTGAAGAAGCCGAACAATTTGCTGAAGAGGATAAGATTCAAAAGGA
RiBIP1_RirG196040             ATGGTTGAAGAAGCCGAACAATTTGCTGAAGAGGATAAGATTCAAAAGGA
BIP1_RirG196040|0|DNA2        ATGGTTGAAGAAGCCGAACAATTTGCTGAAGAGGATAAGATTCAAAAGGA
BIP1_RirG196040|2|N6          ATGGTTGAAGAAGCCGAACAATTTGCTGAAGAGGATAAGATTCAAAAGGA
                              **************************************************

BIP1_RirG196040|1|DNA1        ACGCATAGAAGCTAAACATCAACTTGAAAATTTTGCATACACAATCAAAA
BIP1_RirG196040|3|N33         ACGCATAGAAGCTAAACATCAACTTGAAAATTTTGCATACACAATCAAAA
RiBIP1_RirG196040             ACGCATAGAAGCTAAACATCAACTTGAAAATTTTGCATACACAATCAAAA
BIP1_RirG196040|0|DNA2        ACGCATAGAAGCTAAACATCAACTTGAAAATTTTGCATACACAATCAAAA
BIP1_RirG196040|2|N6          ACGCATAGAAGCTAAACATCAACTTGAAAATTTTGCATACACAATCAAAA
                              **************************************************

BIP1_RirG196040|1|DNA1        GTCAAATTTTTGATGACAGAGGACTTGGTAAGAAAATAAGCGATGACGAC
BIP1_RirG196040|3|N33         GTCAAATTTTTGATGACAGAGGACTTGGTAAGAAAATAAGCGATGACGAC
RiBIP1_RirG196040             GTCAAATTTTTGATGACAGAGGACTTGGTAAGAAAATAAGCGATGACGAC
BIP1_RirG196040|0|DNA2        GTCAAATTTTTGATGACAGAGGACTTGGTAAGAAAATAAGCGATGACGAC
BIP1_RirG196040|2|N6          GTCAAATTTTTGATGACAGAGGACTTGGTAAGAAAATAAGCGATGACGAC
                              **************************************************

BIP1_RirG196040|1|DNA1        AGGAAAATTATTAAAGATTCAATTAAGACGGTTGAAGATTGGTTGAATGA
BIP1_RirG196040|3|N33         AGGAAAATTATTAAAGATTCAATTAAGAC---------------------
RiBIP1_RirG196040             AGGAAAATTATTAAAGATTCAATTAAGACGGTTGAAGATTGGTTGAATGA
BIP1_RirG196040|0|DNA2        AGGAAAATTATTAAAGATTCAATTAAGACGGTTGAAGATTGGTTGAATGA
BIP1_RirG196040|2|N6          AGGAAAATTATTAAAGATTCAATTAAGACGGTTGAAGATTGGTTGAATGA
                              *****************************

BIP1_RirG196040|1|DNA1        TCATTCAAATTCTGCGATCAAGGAAGATTTTGATGAAAAACGGGAAGAAT
BIP1_RirG196040|3|N33         --------------------------------------------------
RiBIP1_RirG196040             TCATTCAAATTCTGCGATCAAGGAAGATTTTGATGAAAAACGGGAAGAAT
BIP1_RirG196040|0|DNA2        TCATTCAAATTCTGCGATCAAGGAAGATTTTGATGAAAAACGGGAAGAAT
BIP1_RirG196040|2|N6          TCATTCAAATTCTGCGATCAAGGAAGATTTTGATGAAAAACGGGAAGAAT

BIP1_RirG196040|1|DNA1        TACAATCTATCGTTAATCCGATAACTACTAAATTGCATGCTGATGGTCAA
BIP1_RirG196040|3|N33         --------------------------------------------------
RiBIP1_RirG196040             TACAATCTATCGTTAATCCGATAACTACTAAATTGCATGCTGATGGTCAA
BIP1_RirG196040|0|DNA2        TACAATCTATCGTTAATCCGATAACTACTAAATTGCATGCTGATGGTCAA
BIP1_RirG196040|2|N6          TACAATCTATCGTTAATCCGATAACTACTAAATTGCATGCTGATGGTCAA

BIP1_RirG196040|1|DNA1        GCTCCACCACCACCAAGGCATGAAGATTTGTAA
BIP1_RirG196040|3|N33         ---------------------------------
RiBIP1_RirG196040             GCTCCACCACCACCAAGGCATGAAGATTTGTAA
BIP1_RirG196040|0|DNA2        GCTCCACCACCACCAAGGCATGAAGATTTGTAA
BIP1_RirG196040|2|N6          GCTCCACCACCACCAAGGCATGAAGATTTGTAA
```

BIP2:

```
BIP2_RirG160690               ATGAAGAAAGCTACTTTCTTCAAATTTATTGCAGTGTTATGTTCACTGTT
BIP2_RirG160690|0|DNA1        ATGAAGAAAGCTACTTTCTTCAAATTTATTGCAGTGTTATGTTCACTGTT
BIP2_RirG160690|1|DNA2        ATGAAGAAAGCTACTTTCTTCAAATTTATTGCAGTGTTATGTTCACTGTT
BIP2_RirG160690|2|N6          ATGAAGAAAGCTACTTTCTTCAAATTTATTGCAGTGTTATGTTCACTGTT
                              **************************************************

BIP2_RirG160690               AATAACTATTCCTATTGTAATAGTAATTGCTGATGAGAAACCCGACGTTG
BIP2_RirG160690|0|DNA1        AATAACTATTCCTATTGTAATAGTAATTGCTGATGAGAAACCCGACGTTG
BIP2_RirG160690|1|DNA2        AATAACTATTCCTATTGTAATAGTAATTGCTGATGAGAAACCCGACGTTG
BIP2_RirG160690|2|N6          AATAACTATTCCTATTGTAATAGTAATTGCTGATGAGAAACCCGACGTTG
                              **************************************************

BIP2_RirG160690               GAACAGGTATAAAAAATTAATTGTTGAATATTGTTCTCTCGAAACTTATAA
BIP2_RirG160690|0|DNA1        GAACAGGTATAAAAAATTAATTGTTGAATATTGTTCTCTCGAAACTTATAA
BIP2_RirG160690|1|DNA2        GAACAGGTATAAAAAATTAATTGTTGAATATTGTTCTCTCGAAACTTATAA
BIP2_RirG160690|2|N6          GAACAGGTATAAAAAATTAATTGTTGAATATTGTTCTCTCGAAACTTATAA
                              **************************************************

BIP2_RirG160690               GTCTAATATTATATTCCATGGATCGACAGTTATCGGTATAGATTTAGGAA
BIP2_RirG160690|0|DNA1        GTCTAATATTATATTCCATGGATCGACAGTTATCGGTATAGATTTAGGAA
BIP2_RirG160690|1|DNA2        GTCTAATATTATATTCCATGGATCGACAGTTATCGGTATAGATTTAGGAA
BIP2_RirG160690|2|N6          GTCTAATATTATATTCCATGGATCGACAGTTATCGGTATAGATTTAGGAA
                              **************************************************

BIP2_RirG160690               CAACTTATTCTTGTGTTGGAGTACACGTAAACGGAAGAGTTGAGATATTG
BIP2_RirG160690|0|DNA1        CAACTTATTCTTGTGTTGGAGTACACGTAAACGGAAGAGTTGAGATATTG
```

```
BIP2_RirG160690|1|DNA2         CAACTTATTCTTGTGTTGGAGTACACGTAAACGGAAGAGTTGAGATATTG
BIP2_RirG160690|2|N6           CAACTTATTCTTGTGTTGGAGTACACGTAAACGGAAGAGTTGAGATATTG
                               **************************************************

BIP2_RirG160690                ACCAATGACCAGGGTAACCGTATCACGCCTTCTTATGTTGCCTTCACCGA
BIP2_RirG160690|0|DNA1         ACCAATGACCAGGGTAACCGTATCACGCCTTCTTATGTTGCCTTCACCGA
BIP2_RirG160690|1|DNA2         ACCAATGACCAGGGTAACCGTATCACGCCTTCTTATGTTGCCTTCACCGA
BIP2_RirG160690|2|N6           ACCAATGACCAGGGTAACCGTATCACGCCTTCTTATGTTGCCTTCACCGA
                               **************************************************

BIP2_RirG160690                TGATGAACGTCTAATTGGCGATGCGGCAAAGAATCAATATTCAAATAACC
BIP2_RirG160690|0|DNA1         TGATGAACGTCTAATTGGCGATGCGGCAAAGAATCAATATTCAAATAACC
BIP2_RirG160690|1|DNA2         TGATGAACGTCTAATTGGCGATGCGGCAAAGAATCAATATTCAAATAACC
BIP2_RirG160690|2|N6           TGATGAACGTCTAATTGGCGATGCGGCAAAGAATCAATATTCAAATAACC
                               **************************************************

BIP2_RirG160690                CTAGGAACACTATTTTTGATGCTAAGCGGCTAATTGGTCGTCGCTTTAGC
BIP2_RirG160690|0|DNA1         CTAGGAACACTATTTTTGATGCTAAGCGGCTAATTGGTCGTCGCTTTAGC
BIP2_RirG160690|1|DNA2         CTAGGAACACTATTTTTGATGCTAAGCGGCTAATTGGTCGTCGCTTTAGC
BIP2_RirG160690|2|N6           CTAGGAACACTATTTTTGATGCTAAGCGGCTAATTGGTCGTCGCTTTAGC
                               **************************************************

BIP2_RirG160690                GATAAAGAAGTTCAACAAGATATTAGGCACTTTCCATTCAAGGTCATTGA
BIP2_RirG160690|0|DNA1         GATAAAGAAGTTCAACAAGATATTAGGCACTTTCCATTCAAGGTCATTGA
BIP2_RirG160690|1|DNA2         GATAAAGAAGTTCAACAAGATATTAGGCACTTTCCATTCAAGGTCATTGA
BIP2_RirG160690|2|N6           GATAAAGAAGTTCAACAAGATATTAGGCACTTTCCATTCAAGGTCATTGA
                               **************************************************

BIP2_RirG160690                CAAAGATGGTAAACCTGTTATTCAAGTAACTGTAAAAGGCGAAGAAAGAA
BIP2_RirG160690|0|DNA1         CAAAGATGGTAAACCTGTTATTCAAGTAACTGTAAAAGGCGAAGAAAGAA
BIP2_RirG160690|1|DNA2         CAAAGATGGTAAACCTGTTATTCAAGTAACTGTAAAAGGCGAAGAAAGAA
BIP2_RirG160690|2|N6           CAAAGATGGTAAACCTGTTATTCAAGTAACTGTAAAAGGCGAAGAAAGAA
                               **************************************************

BIP2_RirG160690                TATTTACTCCTGAAGAAATTTCGGCTATGATTCTTGGTAAAATGAAAGAA
BIP2_RirG160690|0|DNA1         TATTTACTCCTGAAGAAATTTCGGCTATGATTCTTGGTAAAATGAAAGAA
BIP2_RirG160690|1|DNA2         TATTTACTCCTGAAGAAATTTCGGCTATGATTCTTGGTAAAATGAAAGAA
BIP2_RirG160690|2|N6           TATTTACTCCTGAAGAAATTTCGGCTATGATTCTTGGTAAAATGAAAGAA
                               **************************************************

BIP2_RirG160690                ATTGCAGAATCGTATTTAGGCAAGAAGGTTACACATGCAGTAGTTACAGT
BIP2_RirG160690|0|DNA1         ATTGCAGAATCGTATTTAGGCAAGAAGGTTACACATGCAGTAGTTACAGT
BIP2_RirG160690|1|DNA2         ATTGCAGAATCGTATTTAGGCAAGAAGGTTACACATGCAGTAGTTACAGT
BIP2_RirG160690|2|N6           ATTGCAGAATCGTATTTAGGCAAGAAGGTTACACATGCAGTAGTTACAGT
                               **************************************************

BIP2_RirG160690                CCCAGCTTACTTTAATGACGCTCAAAGACAAGCCACAAAAGATGCTGGCG
BIP2_RirG160690|0|DNA1         CCCAGCTTACTTTAATGACGCTCAAAGACAAGCCACAAAAGATGCTGGCG
BIP2_RirG160690|1|DNA2         CCCAGCTTACTTTAATGACGCTCAAAGACAAGCCACAAAAGATGCTGGCG
BIP2_RirG160690|2|N6           CCCAGCTTACTTTAATGACGCTCAAAGACAAGCCACAAAAGATGCTGGCG
                               **************************************************

BIP2_RirG160690                TAATTGCTGGACTTAATGTCCTTCGTATTGTAAATGAACCTACGGCGGCT
BIP2_RirG160690|0|DNA1         TAATTGCTGGACTTAATGTCCTTCGTATTGTAAATGAACCTACGGCGGCT
BIP2_RirG160690|1|DNA2         TAATTGCTGGACTTAATGTCCTTCGTATTGTAAATGAACCTACGGCGGCT
BIP2_RirG160690|2|N6           TAATTGCTGGACTTAATGTCCTTCGTATTGTAAATGAACCTACGGCGGCT
                               **************************************************

BIP2_RirG160690                GCAATTGCATATGGTCTTGATAAATCCGATGGGGAACGTCAGATTCTTGT
BIP2_RirG160690|0|DNA1         GCAATTGCATATGGTCTTGATAAATCCGATGGGGAACGTCAGATTCTTGT
BIP2_RirG160690|1|DNA2         GCAATTGCATATGGTCTTGATAAATCCGATGGGGAACGTCAGATTCTTGT
BIP2_RirG160690|2|N6           GCAATTGCATATGGTCTTGATAAATCCGATGGGGAACGTCAGATTCTTGT
                               **************************************************

BIP2_RirG160690                CTATGATCTTGGTGGTGGTACCTTTGATGTTTCTCTCTTGTCAATTGATG
BIP2_RirG160690|0|DNA1         CTATGATCTTGGTGGTGGTACCTTTGATGTTTCTCTCTTGTCAATTGATG
BIP2_RirG160690|1|DNA2         CTATGATCTTGGTGGTGGTACCTTTGATGTTTCTCTCTTGTCAATTGATG
BIP2_RirG160690|2|N6           CTATGATCTTGGTGGTGGTACCTTTGATGTTTCTCTCTTGTCAATTGATG
                               **************************************************

BIP2_RirG160690                ATGGTGTTTTTGAGGTATTGGCAACTGCCGGCGATACACATTTGGGAGGT
BIP2_RirG160690|0|DNA1         ATGGTGTTTTTGAGGTATTGGCAACTGCCGGCGATACACATTTGGGAGGT
BIP2_RirG160690|1|DNA2         ATGGTGTTTTTGAGGTATTGGCAACTGCCGGCGATACACATTTGGGAGGT
BIP2_RirG160690|2|N6           ATGGTGTTTTTGAGGTATTGGCAACTGCCGGCGATACACATTTGGGAGGT
                               **************************************************
```

```
BIP2_RirG160690             GAAGACTTCGACAACCGAGTTATTGACCACTTCGTTAAACTTTATAAAAA
BIP2_RirG160690|0|DNA1      GAAGACTTCGACAACCGAGTTATTGACCACTTCGTTAAACTTTATAAAAA
BIP2_RirG160690|1|DNA2      GAAGACTTCGACAACCGAGTTATTGACCACTTCGTTAAACTTTATAAAAA
BIP2_RirG160690|2|N6        GAAGACTTCGACAACCGAGTTATTGACCACTTCGTTAAACTTTATAAAAA
                            **************************************************

BIP2_RirG160690             GAAAAATAAGATAGATGTTACACAAGATTTAAAAGCTATGGGTAAATTAA
BIP2_RirG160690|0|DNA1      GAAAAATAAGATAGATGTTACACAAGATTTAAAAGCTATGGGTAAATTAA
BIP2_RirG160690|1|DNA2      GAAAAATAAGATAGATGTTACACAAGATTTAAAAGCTATGGGTAAATTAA
BIP2_RirG160690|2|N6        GAAAAATAAGATAGATGTTACACAAGATTTAAAAGCTATGGGTAAATTAA
                            **************************************************

BIP2_RirG160690             AGCGTGAAGTTGAAAAAGCCAAGCGTACATTATCTTCTCAAATGTCAACT
BIP2_RirG160690|0|DNA1      AGCGTGAAGTTGAAAAAGCCAAGCGTACATTATCTTCTCAAATGTCAACT
BIP2_RirG160690|1|DNA2      AGCGTGAAGTTGAAAAAGCCAAGCGTACATTATCTTCTCAAATGTCAACT
BIP2_RirG160690|2|N6        AGCGTGAAGTTGAAAAAGCCAAGCGTACATTATCTTCTCAAATGTCAACT
                            **************************************************

BIP2_RirG160690             CGTATCGAAATTGAATCATTTCATGATGGTAAAGATTTTTCCGAAACTTT
BIP2_RirG160690|0|DNA1      CGTATCGAAATTGAATCATTTCATGATGGTAAAGATTTTTCCGAAACTTT
BIP2_RirG160690|1|DNA2      CGTATCGAAATTGAATCATTTCATGATGGTAAAGATTTTTCCGAAACTTT
BIP2_RirG160690|2|N6        CGTATCGAAATTGAATCATTTCATGATGGTAAAGATTTTTCCGAAACTTT
                            **************************************************

BIP2_RirG160690             GACCCGTGCCAAATTTGAAGAACTTAATAATGATCTCTTCCGTAAAACAT
BIP2_RirG160690|0|DNA1      GACCCGTGCCAAATTTGAAGAACTTAATAATGATCTCTTCCGTAAAACAT
BIP2_RirG160690|1|DNA2      GACCCGTGCCAAATTTGAAGAACTTAATAATGATCTCTTCCGTAAAACAT
BIP2_RirG160690|2|N6        GACCCGTGCCAAATTTGAAGAACTTAATAATGATCTCTTCCGTAAAACAT
                            **************************************************

BIP2_RirG160690             TAAAGCCCGTTGAACAAGTATTGAAAGATGCAAATATTGATAAGAAGGAT
BIP2_RirG160690|0|DNA1      TAAAGCCCGTTGAACAAGTATTGAAAGATGCAAATATTGATAAGAAGGAT
BIP2_RirG160690|1|DNA2      TAAAGCCCGTTGAACAAGTATTGAAAGATGCAAATATTGATAAGAAGGAT
BIP2_RirG160690|2|N6        TAAAGCCCGTTGAACAAGTATTGAAAGATGCAAATATTGATAAGAAGGAT
                            **************************************************

BIP2_RirG160690             GTACACGATATTGTACTAGTTGGTGGTTCCACACGTATTCCCAAAGTTCA
BIP2_RirG160690|0|DNA1      GTACACGATATTGTACTAGTTGGTGGTTCCACACGTATTCCCAAAGTTCA
BIP2_RirG160690|1|DNA2      GTACACGATATTGTACTAGTTGGTGGTTCCACACGTATTCCCAAAGTTCA
BIP2_RirG160690|2|N6        GTACACGATATTGTACTAGTTGGTGGTTCCACACGTATTCCCAAAGTTCA
                            **************************************************

BIP2_RirG160690             ACAACTCCTTGAAGAATTCTTTAATGGCAAGAAAGCTTCCAAAAACATTA
BIP2_RirG160690|0|DNA1      ACAACTCCTTGAAGAATTCTTTAATGGCAAGAAAGCTTCCAAAAACATTA
BIP2_RirG160690|1|DNA2      ACAACTCCTTGAAGAATTCTTTAATGGCAAGAAAGCTTCCAAAAACATTA
BIP2_RirG160690|2|N6        ACAACTCCTTGAAGAATTCTTTAATGGCAAGAAAGCTTCCAAAAACATTA
                            **************************************************

BIP2_RirG160690             ATCCTGATGAAGCAGTTGCATATGGTGCCGCCATACAAGGAGGTATTCTT
BIP2_RirG160690|0|DNA1      ATCCTGATGAAGCAGTTGCATATGGTGCCGCCATACAAGGAGGTATTCTT
BIP2_RirG160690|1|DNA2      ATCCTGATGAAGCAGTTGCATATGGTGCCGCCATACAAGGAGGTATTCTT
BIP2_RirG160690|2|N6        ATCCTGATGAAGCAGTTGCATATGGTGCCGCCATACAAGGAGGTATTCTT
                            **************************************************

BIP2_RirG160690             TCTGGAGATGAAGATGTAAAGGAAATCCTTTTGGTAGACGTTTGTCCTCT
BIP2_RirG160690|0|DNA1      TCTGGAGATGAAGATGTAAAGGAAATCCTTTTGGTAGACGTTTGTCCTCT
BIP2_RirG160690|1|DNA2      TCTGGAGATGAAGATGTAAAGGAAATCCTTTTGGTAGACGTTTGTCCTCT
BIP2_RirG160690|2|N6        TCTGGAGATGAAGATGTAAAGGAAATCCTTTTGGTAGACGTTTGTCCTCT
                            **************************************************

BIP2_RirG160690             TACACTCGGTATTGAAACAACTGGTGGCGTTATGACAAAATTAATTCCTC
BIP2_RirG160690|0|DNA1      TACACTCGGTATTGAAACAACTGGTGGCGTTATGACAAAATTAATTCCTC
BIP2_RirG160690|1|DNA2      TACACTCGGTATTGAAACAACTGGTGGCGTTATGACAAAATTAATTCCTC
BIP2_RirG160690|2|N6        TACACTCGGTATTGAAACAACTGGTGGCGTTATGACAAAATTAATTCCTC
                            **************************************************

BIP2_RirG160690             GTAACACTGTAATTCCTACTAAGAAGTCACAAATTTTCTCTACCGCCGCG
BIP2_RirG160690|0|DNA1      GTAACACTGTAATTCCTACTAAGAAGTCACAAATTTTCTCTACCGCCGCG
BIP2_RirG160690|1|DNA2      GTAACACTGTAATTCCTACTAAGAAGTCACAAATTTTCTCTACCGCCGCG
BIP2_RirG160690|2|N6        GTAACACTGTAATTCCTACTAAGAAGTCACAAATTTTCTCTACCGCCGCG
                            **************************************************

BIP2_RirG160690             GATAACCAACCTACTGTATTAATTCAAGTTTACGAAGGAGAACGTTCTAT
BIP2_RirG160690|0|DNA1      GATAACCAACCTACTGTATTAATTCAAGTTTACGAAGGAGAACGTTCTAT
BIP2_RirG160690|1|DNA2      GATAACCAACCTACTGTATTAATTCAAGTTTACGAAGGAGAACGTTCTAT
BIP2_RirG160690|2|N6        GATAACCAACCTACTGTATTAATTCAAGTTTACGAAGGAGAACGTTCTAT
                            **************************************************
```

```
BIP2_RirG160690             GACTAAAGATAACAACTTGCTCGGCAAGTTCGAATTAACAGGTATACCGC
BIP2_RirG160690|0|DNA1      GACTAAAGATAACAACTTGCTCGGCAAGTTCGAATTAACAGGTATACCGC
BIP2_RirG160690|1|DNA2      GACTAAAGATAACAACTTGCTCGGCAAGTTCGAATTAACAGGTATACCGC
BIP2_RirG160690|2|N6        GACTAAAGATAACAACTTGCTCGGCAAGTTCGAATTAACAGGTATACCGC
                            **************************************************

BIP2_RirG160690             CTGCACCACGAGGTATACCGCAGATCGAGGTCACATTTGAAATTGATACC
BIP2_RirG160690|0|DNA1      CTGCACCACGAGGTATACCGCAGATCGAGGTCACATTTGAAATTGATACC
BIP2_RirG160690|1|DNA2      CTGCACCACGAGGTATACCGCAGATCGAGGTCACATTTGAAATTGATACC
BIP2_RirG160690|2|N6        CTGCACCACGAGGTATACCGCAGATCGAGGTCACATTTGAAATTGATACC
                            **************************************************

BIP2_RirG160690             AATGGTATAATGAAAGTATCTGCAGCAGATAAAGGTACAGGGAAAACTGA
BIP2_RirG160690|0|DNA1      AATGGTATAATGAAAGTATCTGCAGCAGATAAAGGTACAGGGAAAACTGA
BIP2_RirG160690|1|DNA2      AATGGTATAATGAAAGTATCTGCAGCAGATAAAGGTACAGGGAAAACTGA
BIP2_RirG160690|2|N6        AATGGTATAATGAAAGTATCTGCAGCAGATAAAGGTACAGGGAAAACTGA
                            **************************************************

BIP2_RirG160690             ATCGATTACAATTACAAACGATAAAGGTCGTCTTTCTAAAGAAGAAATTG
BIP2_RirG160690|0|DNA1      ATCGATTACAATTACAAACGATAAAGGTCGTCTTTCTAAAGAAGAAATTG
BIP2_RirG160690|1|DNA2      ATCGATTACAATTACAAACGATAAAGGTCGTCTTTCTAAAGAAGAAATTG
BIP2_RirG160690|2|N6        ATCGATTACAATTACAAACGATAAAGGTCGTCTTTCTAAAGAAGAAATTG
                            **************************************************

BIP2_RirG160690             ATCGTATGGTTGAAGAAGCCGAACAATTTGCTGAAGATGATAAGTTTCAA
BIP2_RirG160690|0|DNA1      ATCGTATGGTTGAAGAAGCCGAACAATTTGCTGAAGATGATAAGTTTCAA
BIP2_RirG160690|1|DNA2      ATCGTATGGTTGAAGAAGCCGAACAATTTGCTGAAGATGATAAGTTTCAA
BIP2_RirG160690|2|N6        ATCGTATGGTTGAAGAAGCCGAACAATTTGCTGAAGATGATAAGTTTCAA
                            **************************************************

BIP2_RirG160690             AAGGAACGCGTAGAATCCAGAAATCAGCTTGAAAATTTTGCATACACAAT
BIP2_RirG160690|0|DNA1      AAGGAACGCGTAGAATCCAGAAATCAGCTTGAAAATTTTGCATACACAAT
BIP2_RirG160690|1|DNA2      AAGGAACGCGTAGAATCCAGAAATCAGCTTGAAAATTTTGCATACACAAT
BIP2_RirG160690|2|N6        AAGGAACGCGTAGAATCCAGAAATCAGCTTGAAAATTTTGCATACACAAT
                            **************************************************

BIP2_RirG160690             CAAAAGTCAAATTTCTGATGACGGAGAACTTGGTAAGAAAATAAGCGATG
BIP2_RirG160690|0|DNA1      CAAAAGTCAAATTTCTGATGACGGAGAACTTGGTAAGAAAATAAGCGATG
BIP2_RirG160690|1|DNA2      CAAAAGTCAAATTTCTGATGACGGAGAACTTGGTAAGAAAATAAGCGATG
BIP2_RirG160690|2|N6        CAAAAGTCAAATTTCTGATGACGGAGAACTTGGTAAGAAAATAAGCGATG
                            **************************************************

BIP2_RirG160690             ACGACAAGAAAACTATTAAAGATGCAATCAAGACGGTTGAAGATTGGTTG
BIP2_RirG160690|0|DNA1      ACGACAAGAAAACTATTAAAGATGCAATCAAGACGGTTGAAGATTGGTTG
BIP2_RirG160690|1|DNA2      ACGACAAGAAAACTATTAAAGATGCAATCAAGACGGTTGAAGATTGGTTG
BIP2_RirG160690|2|N6        ACGACAAGAAAACTATTAAAGATGCAATCAAGACGGTTGAAGATTGGTTG
                            **************************************************

BIP2_RirG160690             AATGACCATTCAATTTCTGCGACCAAGGAAGATTTTGATGAAAAACGGGA
BIP2_RirG160690|0|DNA1      AATGACCATTCAATTTCTGCGACCAAGGAAGATTTTGATGAAAAACGGGA
BIP2_RirG160690|1|DNA2      AATGACCATTCAATTTCTGCGACCAAGGAAGATTTTGATGAAAAACGGGA
BIP2_RirG160690|2|N6        AATGACCATTCAATTTCTGCGACCAAGGAAGATTTTGATGAAAAACGGGA
                            **************************************************

BIP2_RirG160690             AGAATTACAATCAATCGTTAATCCAATAACTACTAAATTGTATGCTGATG
BIP2_RirG160690|0|DNA1      AGAATTACAATCAATCGTTAATCCAATAACTACTAAATTGTATGCTGATG
BIP2_RirG160690|1|DNA2      AGAATTACAATCAATCGTTAATCCAATAACTACTAAATTGTATGCTGATG
BIP2_RirG160690|2|N6        AGAATTACAATCAATCGTTAATCCAATAACTACTAAATTGTATGCTGATG
                            **************************************************

BIP2_RirG160690             GTCAAGCTCCACCTTCACACGAGGAACCACCAAGGCATGAAGATTTGTAA
BIP2_RirG160690|0|DNA1      GTCAAGCTCCACCTTCACACGAGGAACCACCAAGGCATGAAGATTTGTAA
BIP2_RirG160690|1|DNA2      GTCAAGCTCCACCTTCACACGAGGAACCACCAAGGCATGAAGATTTGTAA
BIP2_RirG160690|2|N6        GTCAAGCTCCACCTTCACACGAGGAACCACCAAGGCATGAAGATTTGTAA
                            **************************************************
```

BIP3:

```
BIP3_RirG043980              ATGAAGAAAGCTACTTTCTTTAAATTTATTGCATTGTTATGTTCACTGTT
BIP3_RirG043980|0|DNA2       ATGAAGAAAGCTACTTTCTTTAAATTTATTGCATTGTTATGTTCACTGTT
BIP3_RirG043980|1|DNA1       ATGAAGAAAGCTACTTTCTTTAAATTTATTGCATTGTTATGTTCACTGTT
BIP3_RirG043980|2|N33        ATGAAGAAAGCTACTTTCTTTAAATTTATTGCATTGTTATGTTCACTGTT
BIP3_RirG043980|3|N31        ATGAAGAAAGCTACTTTCTTTAAATTTATTGCATTGTTATGTTCACTGTT
BIP3_RirG043980|5|N36        --------------------------------------------------
BIP3_RirG043980|4|N6         ATGAAGAAAGCTACTTTCTTTAAATTTATTGCATTGTTATGTTCACTGTT

BIP3_RirG043980              AATAACTATTCTTATTGCAAAAGTTATTGTAATTGATGAATCTGACGTTG
BIP3_RirG043980|0|DNA2       AATAACTATTCTTATTGCAAAAGTTATTGTAATTGATGAATCTGACGTTG
BIP3_RirG043980|1|DNA1       AATAACTATTCTTATTGCAAAAGTTATTGTAATTGATGAATCTGACGTTG
BIP3_RirG043980|2|N33        AATAACTATTCTTATTGCAAAAGTTATTGTAATTGATGAATCTGACGTTG
BIP3_RirG043980|3|N31        AATAACTATTCTTATTGCAAAAGTTATTGTAATTGATGAATCTGACGTTG
BIP3_RirG043980|5|N36        --------------------------------------------------
BIP3_RirG043980|4|N6         AATAACTATTCTTATTGCAAAAGTTATTGTAATTGATGAATCTGACGTTG

BIP3_RirG043980              GAACAGGTATAAAATTAATTGCTGAATATTGTTCTTTTGAAACTTATAAG
BIP3_RirG043980|0|DNA2       GAACAGGTATAAAATTAATTGCTGAATATTGTTCTTTTGAAACTTATAAG
BIP3_RirG043980|1|DNA1       GAACAGGTATAAAATTAATTGCTGAATATTGTTCTTTTGAAACTTATAAG
BIP3_RirG043980|2|N33        GAACAGGTATAAAATTAATTGCTGAATATTGTTCTTTTGAAACTTATAAG
BIP3_RirG043980|3|N31        GAACAGGTATAAAATTAATTGCTGAATATTGTTCTTTTGAAACTTATAAG
BIP3_RirG043980|5|N36        --------------------------------------------------
BIP3_RirG043980|4|N6         GAACAGGTATAAAATTAATTGCTGAATATTGTTCTTTTGAAACTTATAAG

BIP3_RirG043980              TTCTAATATTATATCGTCAGTTATCGGTATAGATTTAGGAACAACTTATT
BIP3_RirG043980|0|DNA2       TTCTAATATTATATCGTCAGTTATCGGTATAGATTTAGGAACAACTTATT
BIP3_RirG043980|1|DNA1       TTCTAATATTATATCGTCAGTTATCGGTATAGATTTAGGAACAACTTATT
BIP3_RirG043980|2|N33        TTCTAATATTATATCGTCAGTTATCGGTATAGATTTAGGAACAACTTATT
BIP3_RirG043980|3|N31        TTCTAATATTATATCGTCAGTTATCGGTATAGATTTAGGAACAACTTATT
BIP3_RirG043980|5|N36        --------------------------------------------------
BIP3_RirG043980|4|N6         TTCTAATATTATATCGTCAGTTATCGGTATAGATTTAGGAACAACTTATT

BIP3_RirG043980              CTTGTGTTGGAGTATACGTGAACGGAAGAGTTGAGATATTGACCAATGAT
BIP3_RirG043980|0|DNA2       CTTGTGTTGGAGTATACGTGAACGGAAGAGTTGAGATATTGACCAATGAT
BIP3_RirG043980|1|DNA1       CTTGTGTTGGAGTATACGTGAACGGAAGAGTTGAGATATTGACCAATGAT
BIP3_RirG043980|2|N33        CTTGTGTTGGAGTATACGTGAACGGAAGAGTTGAGATATTGACCAATGAT
BIP3_RirG043980|3|N31        CTTGTGTTGGAGTATACGTGAACGGAAGAGTTGAGATATTGACCAATGAT
BIP3_RirG043980|5|N36        --------------------------------------------------
BIP3_RirG043980|4|N6         CTTGTGTTGGAGTATACGTGAACGGAAGAGTTGAGATATTGACCAATGAT

BIP3_RirG043980              CAGGGTAACCGTATCACGCCTTCTTATGTTGCCTTCACCGATGATGAACG
BIP3_RirG043980|0|DNA2       CAGGGTAACCGTATCACGCCTTCTTATGTTGCCTTCACCGATGATGAACG
BIP3_RirG043980|1|DNA1       CAGGGTAACCGTATCACGCCTTCTTATGTTGCCTTCACCGATGATGAACG
BIP3_RirG043980|2|N33        CAGGGTAACCGTATCACGCCTTCTTATGTTGCCTTCACCGATGATGAACG
BIP3_RirG043980|3|N31        CAGGGTAACCGTATCACGCCTTCTTATGTTGCCTTCACCGATGATGAACG
BIP3_RirG043980|5|N36        --------------------------------------------------
BIP3_RirG043980|4|N6         CAGGGTAACCGTATCACGCCTTCTTATGTTGCCTTCACCGATGATGAACG

BIP3_RirG043980              TTTAATTGGCGATGCGGCAAAGAATCAATATTCAAATAACTCTAAGAACA
BIP3_RirG043980|0|DNA2       TTTAATTGGCGATGCGGCAAAGAATCAATATTCAAATAACTCTAAGAACA
BIP3_RirG043980|1|DNA1       TTTAATTGGCGATGCGGCAAAGAATCAATATTCAAATAACTCTAAGAACA
BIP3_RirG043980|2|N33        TTTAATTGGCGATGCGGCAAAGAATCAATATTCAAATAACTCTAAGAACA
BIP3_RirG043980|3|N31        TTTAATTGGCGATGCGGCAAAGAATCAATATTCAAATAACTCTAAGAACA
BIP3_RirG043980|5|N36        --------------------------------------------------
BIP3_RirG043980|4|N6         TTTAATTGGCGATGCGGCAAAGAATCAATATTCAAATAACTCTAAGAACA

BIP3_RirG043980              CCATTTTTGGTTTTAAGCGACTAATTGGTCGTCGCTTTAGCGATAAAGAG
BIP3_RirG043980|0|DNA2       CCATTTTTGGTTTTAAGCGACTAATTGGTCGTCGCTTTAGCGATAAAGAG
BIP3_RirG043980|1|DNA1       CCATTTTTGGTTTTAAGCGACTAATTGGTCGTCGCTTTAGCGATAAAGAG
BIP3_RirG043980|2|N33        CCATTTTTGGTTTTAAGCGACTAATTGGTCGTCGCTTTAGCGATAAAGAG
BIP3_RirG043980|3|N31        CCATTTTTGGTTTTAAGCGACTAATTGGTCGTCGCTTTAGCGATAAAGAG
BIP3_RirG043980|5|N36        --------------------------------------------------
BIP3_RirG043980|4|N6         CCATTTTTGGTTTTAAGCGACTAATTGGTCGTCGCTTTAGCGATAAAGAG

BIP3_RirG043980              GTTCAACAAGATATTAAGCAGTTTCCATTCAAGGTCATTGACAAAGATGG
BIP3_RirG043980|0|DNA2       GTTCAACAAGATATTAAGCAGTTTCCATTCAAGGTCATTGACAAAGATGG
BIP3_RirG043980|1|DNA1       GTTCAACAAGATATTAAGCAGTTTCCATTCAAGGTCATTGACAAAGATGG
BIP3_RirG043980|2|N33        GTTCAACAAGATATTAAGCAGTTTCCATTCAAGGTCATTGACAAAGATGG
BIP3_RirG043980|3|N31        GTTCAACAAGATATTAAGCAGTTTCCATTCAAGGTCATTGACAAAGATGG
BIP3_RirG043980|5|N36        --------------------------------------------------
BIP3_RirG043980|4|N6         GTTCAACAAGATATTAAGCAGTTTCCATTCAAGGTCATTGACAAAGATGG

BIP3_RirG043980              TAAACCTGTTATTCAAATCACTGTAAAAAGCGAAGAAAGAATATTTACTC
BIP3_RirG043980|0|DNA2       TAAACCTGTTATTCAAATCACTGTAAAAAGCGAAGAAAGAATATTTACTC
BIP3_RirG043980|1|DNA1       TAAACCTGTTATTCAAATCACTGTAAAAAGCGAAGAAAGAATATTTACTC
```

```
BIP3_RirG043980|2|N33          TAAACCTGTTATTCAAATCACTGTAAAAAGCGAAGAAAGAATATTTACTC
BIP3_RirG043980|3|N31          TAAACCTGTTATTCAAATCACTGTAAAAAGCGAAGAAAGAATATTTACTC
BIP3_RirG043980|5|N36          --------------------------------------------------
BIP3_RirG043980|4|N6           TAAACCTGTTATTCAAATCACTGTAAAAAGCGAAGAAAGAATATTTACTC

BIP3_RirG043980                CTGAAGAAATTTCGGCTATGATTCTTGGTAAAATGAAAGAAATTGCAAAA
BIP3_RirG043980|0|DNA2         CTGAAGAAATTTCGGCTATGATTCTTGGTAAAATGAAAGAAATTGCAAAA
BIP3_RirG043980|1|DNA1         CTGAAGAAATTTCGGCTATGATTCTTGGTAAAATGAAAGAAATTGCAAAA
BIP3_RirG043980|2|N33          CTGAAGAAATTTCGGCTATGATTCTTGGTAAAATGAAAGAAATTGCAAAA
BIP3_RirG043980|3|N31          CTGAAGAAATTTCGGCTATGATTCTTGGTAAAATGAAAGAAATTGCAAAA
BIP3_RirG043980|5|N36          --------------------------------------------------
BIP3_RirG043980|4|N6           CTGAAGAAATTTCGGCTATGATTCTTGGTAAAATGAAAGAAATTGCAAAA

BIP3_RirG043980                TCGTATTTAGGCAAGAAAGTTACACATGCAGTAGTTACAGTCCCAGCTTA
BIP3_RirG043980|0|DNA2         TCGTATTTAGGCAAGAAAGTTACACATGCAGTAGTTACAGTCCCAGCTTA
BIP3_RirG043980|1|DNA1         TCGTATTTAGGCAAGAAAGTTACACATGCAGTAGTTACAGTCCCAGCTTA
BIP3_RirG043980|2|N33          TCGTATTTAGGCAAGAAAGTTACACATGCAGTAGTTACAGTCCCAGCTTA
BIP3_RirG043980|3|N31          TCGTATTTAGGCAAGAAAGTTACACATGCAGTAGTTACAGTCCCAGCTTA
BIP3_RirG043980|5|N36          -----------CAAGAAAGTTACACATGCAGTAGTTACAGTCCCAGCTTA
BIP3_RirG043980|4|N6           TCGTATTTAGGCAAGAAAGTTACACATGCAGTAGTTACAGTCCCAGCTTA
                                          ***************************************

BIP3_RirG043980                CTTTAATGACGCTCAAAGACAAGCCACAAAATATGCTGGCGTAATTGCTG
BIP3_RirG043980|0|DNA2         CTTTAATGACGCTCAAAGACAAGCCACAAAATATGCTGGCGTAATTGCTG
BIP3_RirG043980|1|DNA1         CTTTAATGACGCTCAAAGACAAGCCACAAAATATGCTGGCGTAATTGCTG
BIP3_RirG043980|2|N33          CTTTAATGACGCTCAAAGACAAGCCACAAAATATGCTGGCGTAATTGCTG
BIP3_RirG043980|3|N31          CTTTAATGACGCTCAAAGACAAGCCACAAAATATGCTGGCGTAATTGCTG
BIP3_RirG043980|5|N36          CTTTAATGACGCTCAAAGACAAGCCACAAAATATGCTGGCGTAATTGCTG
BIP3_RirG043980|4|N6           CTTTAATGACGCTCAAAGACAAGCCACAAAATATGCTGGCGTAATTGCTG
                               **************************************************

BIP3_RirG043980                GACTTAATGTCCTTCGTATTGTAAATGAACCTACGGAGGCTGCAATTGCA
BIP3_RirG043980|0|DNA2         GACTTAATGTCCTTCGTATTGTAAATGAACCTACGGAGGCTGCAATTGCA
BIP3_RirG043980|1|DNA1         GACTTAATGTCCTTCGTATTGTAAATGAACCTACGGAGGCTGCAATTGCA
BIP3_RirG043980|2|N33          GACTTAATGTCCTTCGTATTGTAAATGAACCTACGGAGGCTGCAATTGCA
BIP3_RirG043980|3|N31          GACTTAATGTCCTTCGTATTGTAAATGAACCTACGGAGGCTGCAATTGCA
BIP3_RirG043980|5|N36          GACTTAATGTCCTTCGTATTGTAAATGAACCTACGGAGGCTGCAATTGCA
BIP3_RirG043980|4|N6           GACTTAATGTCCTTCGTATTGTAAATGAACCTACGGAGGCTGCAATTGCA
                               **************************************************

BIP3_RirG043980                TATGATCTTTATAAATCCGATGGGGAACGTCAGATTCTTGTCTATGATCT
BIP3_RirG043980|0|DNA2         TATGATCTTTATAAATCCGATGGGGAACGTCAGATTCTTGTCTATGATCT
BIP3_RirG043980|1|DNA1         TATGATCTTTATAAATCCGATGGGGAACGTCAGATTCTTGTCTATGATCT
BIP3_RirG043980|2|N33          TATGATCTTTATAAATCCGATGGGGAACGTCAGATTCTTGTCTATGATCT
BIP3_RirG043980|3|N31          TATGATCTTTATAAATCCGATGGGGAACGTCAGATTCTTGTCTATGATCT
BIP3_RirG043980|5|N36          TATGATCTTTATAAATCCGATGGGGAACGTCAGATTCTTGTCTATGATCT
BIP3_RirG043980|4|N6           TATGATCTTTATAAATCCGATGGGGAACGTCAGATTCTTGTCTATGATCT
                               **************************************************

BIP3_RirG043980                TGGTGGTGGTACCTTTGATGTTTCTCTCTTGTCAATCGATGATGGTGTTT
BIP3_RirG043980|0|DNA2         TGGTGGTGGTACCTTTGATGTTTCTCTCTTGTCAATCGATGATGGTGTTT
BIP3_RirG043980|1|DNA1         TGGTGGTGGTACCTTTGATGTTTCTCTCTTGTCAATCGATGATGGTGTTT
BIP3_RirG043980|2|N33          TGGTGGTGGTACCTTTGATGTTTCTCTCTTGTCAATCGATGATGGTGTTT
BIP3_RirG043980|3|N31          TGGTGGTGGTACCTTTGATGTTTCTCTCTTGTCAATCGATGATGGTGTTT
BIP3_RirG043980|5|N36          TGGTGGTGGTACCTTTGATGTTTCTCTCTTGTCAATCGATGATGGTGTTT
BIP3_RirG043980|4|N6           TGGTGGTGGTACCTTTGATGTTTCTCTCTTGTCAATCGATGATGGTGTTT
                               **************************************************

BIP3_RirG043980                TTGAAGTATTGGCAACTGCCGGCGATACACATTTGGGAGGTGAAGACTTC
BIP3_RirG043980|0|DNA2         TTGAAGTATTGGCAACTGCCGGCGATACACATTTGGGAGGTGAAGACTTC
BIP3_RirG043980|1|DNA1         TTGAAGTATTGGCAACTGCCGGCGATACACATTTGGGAGGTGAAGACTTC
BIP3_RirG043980|2|N33          TTGAAGTATTGGCAACTGCCGGCGATACACATTTGGGAGGTGAAGACTTC
BIP3_RirG043980|3|N31          TTGAAGTATTGGCAACTGCCGGCGATACACATTTGGGAGGTGAAGACTTC
BIP3_RirG043980|5|N36          TTGAAGTATTGGCAACTGCCGGCGATACACATTTGGGAGGTGAAGACTTC
BIP3_RirG043980|4|N6           TTGAAGTATTGGCAACTGCCGGCGATACACATTTGGGAGGTGAAGACTTC
                               **************************************************

BIP3_RirG043980                GACAACCGAGTTATTGACTACTTCGTTAAACTTTATAAAAAGAAAAATAA
BIP3_RirG043980|0|DNA2         GACAACCGAGTTATTGACTACTTCGTTAAACTTTATAAAAAGAAAAATAA
BIP3_RirG043980|1|DNA1         GACAACCGAGTTATTGACTACTTCGTTAAACTTTATAAAAAGAAAAATAA
BIP3_RirG043980|2|N33          GACAACCGAGTTATTGACTACTTCGTTAAACTTTATAAAAAGAAAAATAA
BIP3_RirG043980|3|N31          GACAACCGAGTTATTGACTACTTCGTTAAACTTTATAAAAAGAAAAATAA
BIP3_RirG043980|5|N36          GACAACCGAGTTATTGACTACTTCGTTAAACTTTATAAAAAGAAAAATAA
BIP3_RirG043980|4|N6           GACAACCGAGTTATTGACTACTTCGTTAAACTTTATAAAAAGAAAAATAA
                               **************************************************
```

```
BIP3_RirG043980              GGTAGACGTTTCACAAGATTTAAAAGCTATGGATAAATTAAAACGTGAAG
BIP3_RirG043980|0|DNA2       GGTAGACGTTTCACAAGATTTAAAAGCTATGGATAAATTAAAACGTGAAG
BIP3_RirG043980|1|DNA1       GGTAGACGTTTCACAAGATTTAAAAGCTATGGATAAATTAAAACGTGAAG
BIP3_RirG043980|2|N33        GGTAGACGTTTCACAAGATTTAAAAGCTATGGATAAATTAAAACGTGAAG
BIP3_RirG043980|3|N31        GGTAGACGTTTCACAAGATTTAAAAGCTATGGATAAATTAAAACGTGAAG
BIP3_RirG043980|5|N36        GGTAGACGTTTCACAAGATTTAAAAGCTATGGATAAATTAAAACGTGAAG
BIP3_RirG043980|4|N6         GGTAGACGTTTCACAAGATTTAAAAGCTATGGATAAATTAAAACGTGAAG
                             **************************************************

BIP3_RirG043980              TTGAAAAAGCCAAGCGTACATTATCTTCTCAAATGTCAACTCATATCGAA
BIP3_RirG043980|0|DNA2       TTGAAAAAGCCAAGCGTACATTATCTTCTCAAATGTCAACTCATATCGAA
BIP3_RirG043980|1|DNA1       TTGAAAAAGCCAAGCGTACATTATCTTCTCAAATGTCAACTCATATCGAA
BIP3_RirG043980|2|N33        TTGAAAAAGCCAAGCGTACATTATCTTCTCAAATGTCAACTCATATCGAA
BIP3_RirG043980|3|N31        TTGAAAAAGCCAAGCGTACATTATCTTCTCAAATGTCAACTCATATCGAA
BIP3_RirG043980|5|N36        TTGAAAAAGCCAAGCGTACATTATCTTCTCAAATGTCAACTCATATCGAA
BIP3_RirG043980|4|N6         TTGAAAAAGCCAAGCGTACATTATCTTCTCAAATGTCAACTCATATCGAA
                             **************************************************

BIP3_RirG043980              ATTGAATCATTTCATGATGGTAAATATTTTTCCGAAATTTTGACCCGTGC
BIP3_RirG043980|0|DNA2       ATTGAATCATTTCATGATGGTAAATATTTTTCCGAAATTTTGACCCGTGC
BIP3_RirG043980|1|DNA1       ATTGAATCATTTCATGATGGTAAATATTTTTCCGAAATTTTGACCCGTGC
BIP3_RirG043980|2|N33        ATTGAATCATTTCATGATGGTAAATATTTTTCCGAAATTTTGACCCGTGC
BIP3_RirG043980|3|N31        ATTGAATCATTTCATGATGGTAAATATTTTTCCGAAATTTTGACCCGTGC
BIP3_RirG043980|5|N36        ATTGAATCATTTCATGATGGTAAATATTTTTCCGAAATTTTGACCCGTGC
BIP3_RirG043980|4|N6         ATTGAATCATTTCATGATGGTAAATATTTTTCCGAAATTTTGACCCGTGC
                             **************************************************

BIP3_RirG043980              CAAATTTGAAGAACTTAACAATGATCTCTTCCTTAAAACATTAAAGTTCG
BIP3_RirG043980|0|DNA2       CAAATTTGAAGAACTTAACAATGATCTCTTCCTTAAAACATTAAAGTTCG
BIP3_RirG043980|1|DNA1       CAAATTTGAAGAACTTAACAATGATCTCTTCCTTAAAACATTAAAGTTCG
BIP3_RirG043980|2|N33        CAAATTTGAAGAACTTAACAATGATCTCTTCCTTAAAACATTAAAGTTCG
BIP3_RirG043980|3|N31        CAAATTTGAAGAACTTAACAATGATCTCTTCCTTAAAACATTAAAGTTCG
BIP3_RirG043980|5|N36        CAAATTTGAAGAACTTAACAATGATCTCTTCCTTAAAACATTAAAGTTCG
BIP3_RirG043980|4|N6         CAAATTTGAAGAACTTAACAATGATCTCTTCCTTAAAACATTAAAGTTCG
                             **************************************************

BIP3_RirG043980              TTGAACAAGTGTTGAAAGATGCAAATGTTGATAAGAAGGATGTGCACGAT
BIP3_RirG043980|0|DNA2       TTGAACAAGTGTTGAAAGATGCAAATGTTGATAAGAAGGATGTGCACGAT
BIP3_RirG043980|1|DNA1       TTGAACAAGTGTTGAAAGATGCAAATGTTGATAAGAAGGATGTGCACGAT
BIP3_RirG043980|2|N33        TTGAACAAGTGTTGAAAGATGCAAATGTTGATAAGAAGGATGTGCACGAT
BIP3_RirG043980|3|N31        TTGAACAAGTGTTGAAAGATGCAAATGTTGATAAGAAGGATGTGCACGAT
BIP3_RirG043980|5|N36        TTGAACAAGTGTTGAAAGATGCAAATGTTGATAAGAAGGATGTGCACGAT
BIP3_RirG043980|4|N6         TTGAACAAGTGTTGAAAGATGCAAATGTTGATAAGAAGGATGTGCACGAT
                             **************************************************

BIP3_RirG043980              ATTGTACTCGTTGGTGGTTTCACACGTATTCCCAAAGTTCAACAACTCCT
BIP3_RirG043980|0|DNA2       ATTGTACTCGTTGGTGGTTTCACACGTATTCCCAAAGTTCAACAACTCCT
BIP3_RirG043980|1|DNA1       ATTGTACTCGTTGGTGGTTTCACACGTATTCCCAAAGTTCAACAACTCCT
BIP3_RirG043980|2|N33        ATTGTACTCGTTGGTGGTTTCACACGTATTCCCAAAGTTCAACAACTCCT
BIP3_RirG043980|3|N31        ATTGTACTCGTTGGTGGTTTCACACGTATTCCCAAAGTTCAACAACTCCT
BIP3_RirG043980|5|N36        ATTGTACTCGTTGGTGGTTTCACACGTATTCCCAAAGTTCAACAACTCCT
BIP3_RirG043980|4|N6         ATTGTACTCGTTGGTGGTTTCACACGTATTCCCAAAGTTCAACAACTCCT
                             **************************************************

BIP3_RirG043980              TGAAGAATTCTTTAGTGGTAAGAAAACTTTCAAAGATATTAATCCTGATG
BIP3_RirG043980|0|DNA2       TGAAGAATTCTTTAGTGGTAAGAAAACTTTCAAAGATATTAATCCTGATG
BIP3_RirG043980|1|DNA1       TGAAGAATTCTTTAGTGGTAAGAAAACTTTCAAAGATATTAATCCTGATG
BIP3_RirG043980|2|N33        TGAAGAATTCTTTAGTGGTAAGAAAACTTTCAAAGATATTAATCCTGATG
BIP3_RirG043980|3|N31        TGAAGAATTCTTTAGTGGTAAGAAAACTTTCAAAGATATTAATCCTGATG
BIP3_RirG043980|5|N36        TGAAGAATTCTTTAGTGGTAAGAAAACTTTCAAAGATATTAATCCTGATG
BIP3_RirG043980|4|N6         TGAAGAATTCTTTAGTGGTAAGAAAACTTTCAAAGATATTAATCCTGATG
                             **************************************************

BIP3_RirG043980              AAGCAGTTGCATATGGTGCCGCCATACAAGGAGGTATTCTTTCCGGAGAT
BIP3_RirG043980|0|DNA2       AAGCAGTTGCATATGGTGCCGCCATACAAGGAGGTATTCTTTCCGGAGAT
BIP3_RirG043980|1|DNA1       AAGCAGTTGCATATGGTGCCGCCATACAAGGAGGTATTCTTTCCGGAGAT
BIP3_RirG043980|2|N33        AAGCAGTTGCATATGGTGCCGCCATACAAGGAGGTATTCTTTCCGGAGAT
BIP3_RirG043980|3|N31        AAGCAGTTGCATATGGTGCCGCCATACAAGGAGGTATTCTTTCCGGAGAT
BIP3_RirG043980|5|N36        AAGCAGTTGCATATGGTGCCGCCATACAAGGAGGTATTCTTTCCGGAGAT
BIP3_RirG043980|4|N6         AAGCAGTTGCATATGGTGCCGCCATACAAGGAGGTATTCTTTCCGGAGAT
                             **************************************************

BIP3_RirG043980              ATACGGATATACCAATGA
BIP3_RirG043980|0|DNA2       ATACGGATATACCAATGA
BIP3_RirG043980|1|DNA1       ATACGGATATACCAATGA
BIP3_RirG043980|2|N33        ATACGGATATACCAATGA
BIP3_RirG043980|3|N31        ATACGGATATACCAATGA
```

```
BIP3_RirG043980|5|N36         ATACGGATATACCAATGA
BIP3_RirG043980|4|N6          ATACGGATATACCAATGA
                              ******************
```

Figure S3. **Alignments of BIP sequences across the seven *R. irregularis* assemblies.** The *BIP* sequence AJ319763.1 was used to identify the homologous sequences in the assemblies.
