# Supplementary material for: Single Nucleus Genome Sequencing Reveals High Similarity among Nuclei of an Endomycorrhizal Fungus
Source: PLoS Genet. 2014 Jan 9;10(1):e1004078. doi: 10.1371/journal.pgen.1004078 (PMC3886924; doi:10.1371/journal.pgen.1004078)
Supplement: Figure S4 — Alignments of Bg112 sequences across seven R. irregularis assemblies. The Bg112 sequence GU930824.1 was used to identify the homologous sequences in the six assemblies. (PDF) [file pgen.1004078.s004.pdf]

Copy 1 of Bg112:

```
GU930824.1|1|Rir        GATACTGGGAAGTGACCATCAGCTCTTGTCTTACTTTTCTTTGAAGTACGTTTCTTCTTC
GU930824.1|1|DNA1       GATACTGGGAAGTGACCATCAGCTCTTGTCTTACTTTTCTTTGAAGTACGTTTCTTCTTC
GU930824.1|1|N31        GATACTGGGAAGTGACCATCAGCTCTTGTCTTACTTTTCTTTGAAGTACGTTTCTTCTTC
GU930824.1|1|DNA2       GATACTGGGAAGTGACCATCAGCTCTTGTCTTACTTTTCTTTGAAGTACGTTTCTTCTTC
                        ************************************************************

GU930824.1|1|Rir        TTTTTACCTTTCTTCTCACTATTCTCTTTATTATTATTATTATTATTATTATTATTATTA
GU930824.1|1|DNA1       TTTTTACCTTTCTTCTCACTATTCTCTTTATTATTATTATTATTATTATTATTATTATTA
GU930824.1|1|N31        TTTTTACCTTTCTTCTCACTATTCTCTTTATTATTATTATTATTATTATTATTATTATTA
GU930824.1|1|DNA2       TTTTTACCTTTCTTCTCACTATTCTCTTTATTATTATTATTATTATTATTATTATTATTA
                        ************************************************************

GU930824.1|1|Rir        AGATTATGAATATCTTCATCAGATGAATAAATATGAGGATCGGAGAATTTGATACGACC
GU930824.1|1|DNA1       AGATTATGAATATCTTCATCAGATGAATAAATATGAGGATCGGAGAATTTGATACGACC
GU930824.1|1|N31        AGATTATGAATATCTTCATCAGATGAATAAATATGAGGATCGGAGAATTTGATACGACC
GU930824.1|1|DNA2       AGATTATGAATATCTTCATCAGATGAATAAATATGAGGATCGGAGAATTTGATACGACC
                        ***********************************************************
```

Copy 2 of Bg112:

```
GU930824.1|3|Rir        GAAACTGGGAAGTGACCATCAGCTCTTATTGAAGTACGTTTCTTCTTCTTTTTACCTTTC
GU930824.1|2|N6         GAAACTGGGAAGTGACCATCAGCTCTTATTGAAGTACGTTTCTTCTTCTTTTTACCTTTC
GU930824.1|2|DNA1       GAAACTGGGAAGTGACCATCAGCTCTTATTGAAGTACGTTTCTTCTTCTTTTTACCTTTC
GU930824.1|2|DNA2       GAAACTGGGAAGTGACCATCAGCTCTTATTGAAGTACGTTTCTTCTTCTTTTTACCTTTC
GU930824.1|2|N33        GAAACTGGGAAGTGACCATCAGCTCTTATTGAAGTACGTTTCTTCTTCTTTTTACCTTTC
GU930824.1|2|N31        GAAACTGGGAAGTGACCATCAGCTCTTATTGAAGTACGTTTCTTCTTCTTTTTACCTTTC
                        ************************************************************

GU930824.1|3|Rir        TTCTCACTATTCTCTTTATTATTATTATTATTATTATTATTATTATTAAGATTATGAATA
GU930824.1|2|N6         TTCTCACTATTCTCTTTATTATTATTATTATTATTATTATTATTATTAAGATTATGAATA
GU930824.1|2|DNA1       TTCTCACTATTCTCTTTATTATTATTATTATTATTATTATTATTATTAAGATTATGAATA
GU930824.1|2|DNA2       TTCTCACTATTCTCTTTATTATTATTATTATTATTATTATTATTATTAAGATTATGAATA
GU930824.1|2|N33        TTCTCACTATTCTCTTTATTATTATTATTATTATTATTATTATTATTAAGATTATGAATA
GU930824.1|2|N31        TTCTCACTATTCTCTTTATTATTATTATTATTATTATTATTATTATTAAGATTATGAATA
                        ************************************************************

GU930824.1|3|Rir        TCTTCATCAGATGAATAAATATGAGGATCGGAGAATTTGATATGACCT
GU930824.1|2|N6         TCTTCATCAGATGAATAAATATGAGGATCGGAGAATTTGATATGACCT
GU930824.1|2|DNA1       TCTTCATCAGATGAATAAATATGAGGATCGGAGAATTTGATATGACCT
GU930824.1|2|DNA2       TCTTCATCAGATGAATAAATATGAGGATCGGAGAATTTGATATGACCT
GU930824.1|2|N33        TCTTCATCAGATGAATAAATATGAGGATCGGAGAATTTGATATGACCT
GU930824.1|2|N31        TCTTCATCAGATGAATAAATATGAGGATCGGAGAATTTGATATGACCT
                        ************************************************
```

Copy 3 of Bg112:

```
GU930824.1|3|Rir        GAAACCGGGAAGTGACCATCAGCTCTTGTCTTACTTTTCATTGAAGTACGTTTCTTCTTC
GU930824.1|3|N6         GAAACCGGGAAGTGACCATCAGCTCTTGTCTTACTTTTCATTGAAGTACGTTTCTTCTTC
GU930824.1|3|DNA1       GAAACCGGGAAGTGACCATCAGCTCTTGTCTTACTTTTCATTGAAGTACGTTTCTTCTTC
GU930824.1|3|DNA2       GAAACCGGGAAGTGACCATCAGCTCTTGTCTTACTTTTCATTGAAGTACGTTTCTTCTTC
GU930824.1|3|N36        GAAACCGGGAAGTGACCATCAGCTCTTGTCTTACTTTTCATTGAAGTACGTTTCTTCTTC
GU930824.1|3|N31        GAAACCGGGAAGTGACCATCAGCTCTTGTCTTACTTTTCATTGAAGTACGTTTCTTCTTC
                        ************************************************************

GU930824.1|3|Rir        TTTTTACCTTTCTTCTCACTATACTCTTTATTATTATTATTAAGATTATAAATATCTTCA
GU930824.1|3|N6         TTTTTACCTTTCTTCTCACTATACTCTTTATTATTATTATTAAGATTATAAATATCTTCA
GU930824.1|3|DNA1       TTTTTACCTTTCTTCTCACTATACTCTTTATTATTATTATTAAGATTATAAATATCTTCA
GU930824.1|3|DNA2       TTTTTACCTTTCTTCTCACTATACTCTTTATTATTATTATTAAGATTATAAATATCTTCA
GU930824.1|3|N36        TTTTTACCTTTCTTCTCACTATACTCTTTATTATTATTATTAAGATTATAAATATCTTCA
GU930824.1|3|N31        TTTTTACCTTTCTTCTCACTATACTCTTTATTATTATTATTAAGATTATAAATATCTTCA
                        ************************************************************

GU930824.1|3|Rir        TCAGATGAATAAATATGAGGATTGGAGAATTTGATATGACCT
GU930824.1|3|N6         TCAGATGAATAAATATGAGGATTGGAGAATTTGATATGACCT
GU930824.1|3|DNA1       TCAGATGAATAAATATGAGGATTGGAGAATTTGATATGACCT
GU930824.1|3|DNA2       TCAGATGAATAAATATGAGGATTGGAGAATTTGATATGACCT
GU930824.1|3|N36        TCAGATGAATAAATATGAGGATTGGAGAATTTGATATGACCT
GU930824.1|3|N31        TCAGATGAATAAATATGAGGATTGGAGAATTTGATATGACCT
                        ******************************************
```

Figure S4. **Alignments of *Bg112* sequences across seven *R. irregularis* assemblies.** The *Bg112* sequence GU930824.1 was used to identify the homologous sequences in the six assemblies.
