# Supplementary material for: Single Nucleus Genome Sequencing Reveals High Similarity among Nuclei of an Endomycorrhizal Fungus
Source: PLoS Genet. 2014 Jan 9;10(1):e1004078. doi: 10.1371/journal.pgen.1004078 (PMC3886924; doi:10.1371/journal.pgen.1004078)
Supplement: Table S1 — Summary of the sequenced read data. (DOCX) [file pgen.1004078.s008.docx]

| **Sample type** | **Insert Size** | **Total Data (GB)** | **Data after filtering (GB)** | **Sequence coverage (x)** | **Read Length** |
| --- | --- | --- | --- | --- | --- |
| N31 | 350bp | 4.0 | 3.5 | 28 | 90 |
| N33 | 350bp | 4.0 | 3.4 | 28 | 90 |
| N36 | 350bp | 3.7 | 3.2 | 26 | 90 |
| DNA1 | 350bp | 2.9 | 2.6 | 20 | 90 |
| DNA2 | 350bp | 3.7 | 3.4 | 26 | 90 |
| N6 | 350bp | 3.2 | 2.9 | 22 | 90 |

**Table S1. Summary of the sequenced read data.**
