# Supplementary material for: Single Nucleus Genome Sequencing Reveals High Similarity among Nuclei of an Endomycorrhizal Fungus
Source: PLoS Genet. 2014 Jan 9;10(1):e1004078. doi: 10.1371/journal.pgen.1004078 (PMC3886924; doi:10.1371/journal.pgen.1004078)
Supplement: Table S2 — Overview of SNPs and INDELs in each sample identified by mapping its reads onto the reference genome. (DOCX) [file pgen.1004078.s009.docx]

**Table S2.** **Overview of SNPs and INDELs in each sample identified by mapping its reads onto the reference genome.**

**Sample name SNPs INDELS**

**__________________________________________________________________________________________________________**

Nucleus N06 11,647 5,156

Nucleus N31 8,547 4,765

Nucleus N33 9,676 5,156

Nucleus N36 9,593 4,747

DNA1 8,304 5,236

DNA2 7,215 4,765

**__________________________________________________________________________________________________________**
