# Supplementary material for: Single Nucleus Genome Sequencing Reveals High Similarity among Nuclei of an Endomycorrhizal Fungus
Source: PLoS Genet. 2014 Jan 9;10(1):e1004078. doi: 10.1371/journal.pgen.1004078 (PMC3886924; doi:10.1371/journal.pgen.1004078)
Supplement: Table S4 — Summary of the core eukaryotic genes (CEG) in the assembly. (DOCX) [file pgen.1004078.s011.docx]

**Table S4. Summary of the core eukaryotic genes (CEG) in the assembly.**

|  | Number of CEG genes | Number of assembled genes | Percentage (%) |
| --- | --- | --- | --- |
| Group 1 | 66 | 58 | 87.9 |
| Group 2 | 56 | 53 | 94.6 |
| Group 3 | 61 | 56 | 91.8 |
| Group 4 | 65 | 62 | 95.4 |
| Total | 248 | 229 | 92.3 |
