# Supplementary material for: Single Nucleus Genome Sequencing Reveals High Similarity among Nuclei of an Endomycorrhizal Fungus
Source: PLoS Genet. 2014 Jan 9;10(1):e1004078. doi: 10.1371/journal.pgen.1004078 (PMC3886924; doi:10.1371/journal.pgen.1004078)
Supplement: Table S8 — Summary of top ten Interpro domains in the annotated unique R. irregularis proteins. (DOCX) [file pgen.1004078.s015.docx]

| AC | Pfam_ID | # proteins | Description |
| --- | --- | --- | --- |
| PF07714 | Pkinase_Tyr | 1,714 | Molecular Function: protein kinase activity (GO:0004672);  Molecular Function: ATP binding (GO:0005524);  Biological Process: protein phosphorylation (GO:0006468) |
| PF00651 | BTB | 1,076 | Molecular Function: protein binding (GO:0005515) |
| PF08238 | Sel1 | 921 | Sel1-like |
| PF00069 | Pkinase | 795 | Molecular Function: protein kinase activity (GO:0004672);  Molecular Function: ATP binding (GO:0005524);  Biological Process: protein phosphorylation (GO:0006468) |
| PF07534 | TLD | 793 | NULL |
| PF07707 | BACK | 502 | BTB/Kelch-associated |
| PF00078 | RVT_1 | 254 | Molecular Function: RNA binding (GO:0003723);  Molecular Function: RNA-directed DNA polymerase activity (GO:0003964);  Biological Process: RNA-dependent DNA replication (GO:0006278) |
| PF00076 | RRM_1 | 192 | Molecular Function: nucleic acid binding (GO:0003676) |
| PF00075 | RnaseH | 189 | Molecular Function: nucleic acid binding (GO:0003676);  Molecular Function: ribonuclease H activity (GO:0004523) |
| PF00400 | WD40 | 188 | WD40 repeat; subgroup |

**Table S8. Summary of top ten Interpro domains in the annotated unique *R. irregularis* proteins.**
