# Supplementary material for: Single Nucleus Genome Sequencing Reveals High Similarity among Nuclei of an Endomycorrhizal Fungus
Source: PLoS Genet. 2014 Jan 9;10(1):e1004078. doi: 10.1371/journal.pgen.1004078 (PMC3886924; doi:10.1371/journal.pgen.1004078)
Supplement: Table S9 — Classification and distribution of repeats based on Repbase TE library. (DOCX) [file pgen.1004078.s016.docx]

**Table S9. Classification and distribustion of repeats based on Repbase TE library.**

| **Type** | **SubType** | **Copy number** | **Length (bp)** | **% of the genome** |
| --- | --- | --- | --- | --- |
| **DNA transposon** |  |  |  |  |
|  | Academ | 25 | 1323 | 0.000938274 |
|  | CMC-Chapaev | 128 | 7881 | 0.005589222 |
|  | CMC-Chapaev-3 | 14 | 869 | 0.000616297 |
|  | CMC-EnSpm | 1462 | 137379 | 0.097429478 |
|  | CMC-Mirage | 1 | 41 | 2.91E-05 |
|  | CMC-Transib | 69 | 5282 | 0.003746006 |
|  | Chapaev | 2 | 124 | 8.79E-05 |
|  | Crypton | 3 | 234 | 0.000165953 |
|  | En-Spm | 3 | 168 | 0.000119146 |
|  | Ginger | 177 | 18781 | 0.013319525 |
|  | Helitron | 451 | 90893 | 0.064461508 |
|  | Kolobok | 2 | 95 | 6.74E-05 |
|  | Kolobok-Hydra | 17 | 1717 | 0.0012177 |
|  | Kolobok-T2 | 31 | 2526 | 0.001791445 |
|  | MULE-F | 2 | 104 | 7.38E-05 |
|  | MULE-MuDR | 694 | 83734 | 0.05938433 |
|  | MULE-NOF | 8 | 543 | 0.000385097 |
|  | Maverick | 1006 | 158073 | 0.112105706 |
|  | Merlin | 10 | 985 | 0.000698564 |
|  | MuDR | 13 | 1313 | 0.000931182 |
|  | Novosib | 4 | 285 | 0.000202123 |
|  | P | 174 | 13460 | 0.009545861 |
|  | P-Fungi | 45 | 5986 | 0.004245284 |
|  | PIF-Harbinger | 179 | 13788 | 0.009778479 |
|  | PIF-ISL2EU | 12 | 733 | 0.000519845 |
|  | PiggyBac | 34 | 8465 | 0.006003396 |
|  | Sola | 78 | 5271 | 0.003738204 |
|  | TcMar | 32 | 2321 | 0.001646058 |
|  | TcMar-Ant1 | 40 | 5443 | 0.003860187 |
|  | TcMar-Fot1 | 124 | 9584 | 0.006796993 |
|  | TcMar-ISRm11 | 21 | 1300 | 0.000921963 |
|  | TcMar-Mariner | 13 | 693 | 0.000491477 |
|  | TcMar-Mogwai | 1 | 46 | 3.26E-05 |
|  | TcMar-Pogo | 217 | 38566 | 0.027351089 |
|  | TcMar-Sagan | 37 | 2961 | 0.002099947 |
|  | TcMar-Stowaway | 18 | 1236 | 0.000876574 |
|  | TcMar-Tc1 | 420 | 65672 | 0.046574721 |
|  | TcMar-Tc2 | 127 | 31104 | 0.022059023 |
|  | TcMar-Tc4 | 2 | 87 | 6.17E-05 |
|  | TcMar-Tigger | 133 | 15693 | 0.011129509 |
|  | TcMar-m44 | 4 | 289 | 0.000204959 |
|  | Transib | 2 | 183 | 0.000129784 |
|  | Unknown | 1238 | 131708 | 0.093407593 |
|  | Zator | 128 | 30038 | 0.021303013 |
|  | hAT | 178 | 12958 | 0.009189841 |
|  | hAT-Ac | 1067 | 91669 | 0.065011849 |
|  | hAT-Blackjack | 118 | 7269 | 0.00515519 |
|  | hAT-Charlie | 67 | 4123 | 0.00292404 |
|  | hAT-Gulliver | 2 | 144 | 0.000102125 |
|  | hAT-Pegasus | 4 | 160 | 0.000113472 |
|  | hAT-Restless | 4 | 328 | 0.000232618 |
|  | hAT-Tag1 | 260 | 19433 | 0.013781925 |
|  | hAT-Tip100 | 138 | 11297 | 0.008011856 |
|  | hAT-Tol2 | 6 | 436 | 0.000309212 |
|  | hAT-hAT1 | 4 | 424 | 0.000300702 |
|  | hAT-hAT5 | 6 | 432 | 0.000306375 |
|  | hAT-hATm | 83 | 5540 | 0.00392898 |
|  | hAT-hATw | 22 | 1413 | 0.001002103 |
|  | hAT-hATx | 14 | 831 | 0.000589347 |
|  | hAT-hobo | 43 | 4101 | 0.002908438 |
| **LINE** |  |  |  |  |
|  | Ambal | 15 | 1077 | 0.000763811 |
|  | CR1 | 117 | 9140 | 0.006482107 |
|  | CR1-Zenon | 4 | 181 | 0.000128366 |
|  | DRE | 48 | 4281 | 0.003036094 |
|  | Dong-R4 | 21 | 1496 | 0.001060966 |
|  | I | 89 | 5721 | 0.004057345 |
|  | Jockey | 158 | 19885 | 0.014102484 |
|  | L1 | 330 | 24619 | 0.017459847 |
|  | L1-Tx1 | 29 | 1809 | 0.001282947 |
|  | L2 | 302 | 24617 | 0.017458429 |
|  | L2-Hydra | 1 | 46 | 3.26E-05 |
|  | LOA | 12 | 607 | 0.000430486 |
|  | Odin | 1 | 57 | 4.04E-05 |
|  | Penelope | 287 | 30106 | 0.021351239 |
|  | Proto1 | 32 | 2641 | 0.001873003 |
|  | Proto2 | 2 | 131 | 9.29E-05 |
|  | R1 | 64 | 6446 | 0.004571517 |
|  | R2 | 162 | 17835 | 0.01264862 |
|  | R2-Hero | 2 | 91 | 6.45E-05 |
|  | RTE | 1 | 64 | 4.54E-05 |
|  | RTE-BovB | 25 | 1444 | 0.001024088 |
|  | RTE-RTE | 2 | 223 | 0.000158152 |
|  | RTE-X | 11 | 650 | 0.000460981 |
|  | Rex-Babar | 13 | 855 | 0.000606368 |
|  | Tad1 | 18 | 2161 | 0.001532586 |
|  | Unknown | 4 | 231 | 0.000163826 |
|  | telomeric | 1 | 69 | 4.89E-05 |
| **LTR** |  |  |  |  |
|  | Caulimovirus | 13 | 1169 | 0.000829057 |
|  | Copia | 946 | 81148 | 0.057550333 |
|  | DIRS | 58 | 6986 | 0.004954486 |
|  | ERV-Lenti | 1 | 71 | 5.04E-05 |
|  | ERV1 | 178 | 10637 | 0.007543783 |
|  | ERVK | 96 | 5779 | 0.004098479 |
|  | ERVL | 15 | 926 | 0.000656721 |
|  | ERVL-MaLR | 3 | 404 | 0.000286518 |
|  | Gypsy | 2129 | 348886 | 0.24743069 |
|  | Ngaro | 61 | 7116 | 0.005046682 |
|  | Pao | 261 | 22656 | 0.016067683 |
|  | Unknown | 54 | 4017 | 0.002848865 |
|  | Viper | 2 | 127 | 9.01E-05 |
| **SINE** |  |  |  |  |
|  | 5S | 13 | 1448 | 0.001026925 |
|  | 7SL | 1 | 74 | 5.25E-05 |
|  | Alu | 3 | 381 | 0.000270206 |
|  | B4 | 2 | 124 | 8.79E-05 |
|  | C | 2 | 152 | 0.000107799 |
|  | Deu | 1 | 65 | 4.61E-05 |
|  | ID | 7 | 533 | 0.000378005 |
|  | Salmon | 3 | 174 | 0.000123401 |
|  | Unknown | 2 | 192 | 0.000136167 |
|  | V | 1 | 63 | 4.47E-05 |
|  | tRNA-CR1 | 7 | 386 | 0.000273752 |
|  | tRNA-Glu | 3 | 206 | 0.000146096 |
|  | tRNA-Lys | 6 | 429 | 0.000304248 |
|  | tRNA-RTE | 1 | 65 | 4.61E-05 |
| **Total** |  | **14837** | **1570779** | **1.114** |
